# Psychometric Properties of Pain Scales in Inpatient Settings: An Umbrella Review

**DOI:** 10.1111/jocn.70071

**Published:** 2025-08-18

**Authors:** Fabio D'Agostino, Noemi Giannetta, Gianfranco Sanson, Claudia Fantuzzi, Sara Cignola, Manuele Cesare, Daniela D'Angelo, Antonello Cocchieri

**Affiliations:** ^1^ Department of Medicine Saint Camillus International University of Health Sciences Rome Italy; ^2^ Department of Medicine Surgery and Health Sciences, University of Trieste Trieste Italy; ^3^ Department of Biomedicine and Prevention University of Rome Tor Vergata Rome Italy; ^4^ Section of Hygiene, Department of Life Sciences and Public Health Catholic University of the Sacred Heart Rome Italy; ^5^ A. Gemelli IRCCS University Hospital Foundation Rome Italy; ^6^ Centro Nazionale Clinical Governance Ed Eccellenza Delle Cure Istituto Superiore di Sanità Rome Italy

**Keywords:** inpatients, pain measurement, psychometrics, systematic review, umbrella review

## Abstract

**Aims:**

To identify the pain assessment scales with the best psychometric properties to be used by nurses in an inpatient setting.

**Design:**

Umbrella review.

**Methods:**

A comprehensive search of four databases was conducted for systematic reviews published from July 2013 to November 2024, focusing on psychometric properties of pain scales used in inpatient settings. Inclusion criteria required scales to assess subjective or behavioural pain and be nurse‐administered, while reviews without detailed psychometric data were excluded. Screening, quality appraisal (JBI checklist), and data extraction were performed independently by two researchers. Data synthesis combined qualitative and quantitative approaches, with psychometric properties evaluated using the COSMIN checklist. The study was reported in accordance with the Preferred Reporting Items for Overviews of Reviews (PRIOR) statement.

**Results:**

Seventeen articles met the inclusion criteria, identifying 41 scales used across various patient populations, including critical care, paediatric, postoperative, cancer, cerebral palsy, disorders of consciousness, low back and neck pain, stroke and verbal communication disorders. The Paediatric Pain Profile, the Breakthrough Pain Assessment Tool and the Questionnaire on Pain caused by Spasticity demonstrated adequate psychometric properties, although the positive findings for the latter two should be confirmed by at least one additional study. Most of the scales (*n* = 36) require further studies to validate their use in clinical practice. For two scales, their clinical use remains questionable.

**Conclusion:**

The Paediatric Pain Profile, the Breakthrough Pain Assessment Tool, and the Questionnaire on Pain caused by Spasticity can be recommended for use. Unidimensional scales should complement, rather than replace, multidimensional scales to ensure a comprehensive pain assessment. Standardising documentation with validated scales enhances clinical decision‐making, care quality, research usability, and reduces documentation burden.

**Summary**

- What does this paper contribute to the wider global clinical community?
  ○ In this paper, we identified 41 pain assessment scales used in inpatient settings and evaluated their psychometric properties through an umbrella review of 17 systematic reviews.
  ○ We found that the Paediatric Pain Profile, the Breakthrough Pain Assessment Tool, and the Questionnaire on Pain caused by Spasticity are the only scales currently recommended for use.
  ○ We highlighted that, despite their widespread use, the clinical applicability of most of the identified scales remains questionable due to variability in psychometric quality, underscoring the need for further validation before their routine adoption in clinical practice.

## 1 Introduction

Worldwide, pain is a daily experience for billions of people of all ages, having different pathogenesis, acute or chronic course, and resulting in a high demand for health services. Pain is a major global health concern, with a deep impact not only on individual citizens but on the whole society from a clinical, economic and social point of view (Henschke et al. 2015). A multicenter study estimated an overall weighted age and sex‐standardised 27.5% prevalence of pain in 52 countries, being women, older people, and rural residents significantly more likely to report pain (Zimmer et al. 2022).

Defining pain is neither simple nor obvious. Indeed, pain is a multifaceted sensation arising from the interaction between unpleasant stimuli produced by the peripheral nervous system and cognitive and affective processes (Garland 2012). Accordingly, pain is defined as ‘an unpleasant sensory and emotional experience associated with, or resembling that associated with, actual or potential tissue damage’ (Raja et al. 2020).

A reliable pain assessment is critical, as it allows an objective pain diagnosis and monitoring over time, also in relation to the effectiveness of the provided treatments. However, assessing pain is challenging since it is a subjective and complex experience inaccessible to external observers (De Ruddere and Tait 2018). Therefore, pain evaluation relies primarily on patient self‐reporting using validated tools. For individuals unable to self‐report (e.g., cognitive impairment, newborn/infant age) (Schofield and Gnass 2023), behavioural tools based on observable pain indicators have been developed.

Nurses play a pivotal role in assessing and managing pain, as stated in many nurses' professional codes of ethics (Angeletti et al. 2021). In the hospital setting, pain occurs with a high prevalence (ranging from 20% to 70%) and is often associated with medical invasive procedures or pre‐existing chronic conditions (Das et al. 2020; Notaro et al. 2021; Wu et al. 2020). Therefore, it is crucial to monitor pain throughout a patient's stay using valid and reliable pain scales (Karcioglu et al. 2018). Furthermore, the widespread use of electronic health records has facilitated the development of information models for specific phenomena like pain, by relying on standardised data and validated scales to enhance both primary and secondary data utilisation (Gaedke Nomura et al. 2021; Westra et al. 2018). To date, extensive research has been conducted to test the many pain scales, resulting in significant progress in their use (Schofield and Gnass 2023), which results have been analysed and compared through several systematic literature reviews.

The umbrella review methodology enables the synthesis of evidence from multiple systematic reviews addressing the same topic across different conditions, populations, or settings (Higgins et al. 2019). An umbrella review of pain scales used across different patient populations is useful to provide a synthesis of widely adopted and validated instruments for hospitalised patients, supporting their selection for integration into pain information models. Therefore, we conducted an umbrella review with the following aims: (a) describing the pain scales used for hospitalised patients; (b) reporting their characteristics (e.g., psychometric properties, usability); and (c) identifying the scales with the best psychometric properties and recommendations for use.

## 2 Methods

After its registration in the International Prospective Register of Systematic Reviews (PROSPERO) (No. CRD42023447507), the study protocol was changed from a rapid to an umbrella review upon realising that numerous systematic reviews were already published on the topic. The study was reported in accordance with the Preferred Reporting Items for Overviews of Reviews (PRIOR) statement (Gates et al. 2022) (Appendix).

To formulate the research question, we involved two librarians of Trieste University, considering: all hospitalised patients (e.g., adults, paediatric) as Population; pain assessment as Intervention; psychometric properties of the scale as Outcome. Literature published between July 2013 and November 2024 was searched on Cochrane Database of Systematic Reviews, CINAHL, PsycInfo, and PubMed MEDLINE databases (Table 1). Only systematic reviews written in English, addressing at least one psychometric property of the scales (e.g., validity, reliability, responsiveness, sensitivity, specificity) and evaluating them through appropriate quality appraisal tools (e.g., the COSMIN checklist) (Mokkink et al. 2018) were considered for inclusion. Reviews providing incomplete psychometric data, as well as study protocols and guidelines, were excluded. Only scales measuring an individual's subjective pain experience or physiological/behavioural cues (i.e., single‐dimensional pain measures or multidimensional pain assessments) were included, if suitable for use in hospital or clinical settings, regardless of patients' health conditions or age. Scales unusable by nurses (e.g., for physicians or paramedics) or those assessing functional limitations, quality of life, or pain‐related disability were excluded.

**TABLE 1:** Search strategies used in the four bibliographic databases.

**PsycInfo**
( (TI pain OR “pain assessment” OR “pain scale” OR “pain tool” OR “pain assessment tool” OR “pain instrument” OR “pain recognition” OR “pain score” OR “pain measurement”)) AND ( reproducibility OR validity OR reliability OR psychometric OR valid OR reliable OR sensibility OR accuracy OR responsiveness OR “predictive value*” OR specificity OR simplicity ) AND ( ( MR ( “systematic review” OR “meta‐analysis” ) AND LA english ) )
**PubMed Medline**
(“hospitalization”[MeSH Terms] OR “hospitalization”[All Fields] OR “hospitalisation”[All Fields] OR “hospitals”[MeSH Terms] OR “hospital”[All Fields] OR “inpatients”[MeSH Terms] OR “inpatients”[All Fields] OR “inpatient”[All Fields]) AND (((“Reproducibility of Results”[Mesh] OR “Dimensional Measurement Accuracy”[Mesh] OR “Sensitivity and Specificity”[Mesh] OR validity OR reliability OR psychometric OR valid OR reliable OR sensibility OR accuracy OR responsiveness OR “predictive value*” OR specificity OR simplicity) AND (“Pain Measurement”[Mesh] OR pain[Title] OR “pain assessment” OR “pain scale” OR “pain tool” OR “pain assessment tool” OR “pain instrument”)) AND (“systematic review”[Publication Type] OR meta‐analysis[Publication Type] OR “meta analysis”[Publication Type])) AND (english[Language])
**CINAHL**
( ( ( TX “hospitalization” OR TX “hospitalisation” OR TX “hospital” OR TX “inpatients” OR TX “inpatient” ) OR (MH “Hospitalization”) OR (MH “Hospitals”) OR (MH “Inpatients”) ) AND ( ( TX “validity” OR TX “reliability” OR TX “psychometric” OR TX “valid” OR TX “reliable” OR TX “sensibility” OR TX “accuracy” OR TX “responsiveness” OR TX “predictive value*” OR TX “specificity” OR TX “simplicity” ) OR ( (MH “Sensitivity and Specificity”) ) OR (MH “Reliability”) OR (MH “Validity”) OR (MH “Reproducibility of Results”) ) AND ( ( TI pain OR TX “pain assessment” OR TX “pain scale” OR TX “pain tool” OR TX “pain assessment tool” OR TX “pain instrument” ) OR (MH “Pain Measurement”) ) ) AND ( PT systematic review OR PT meta‐analysis OR PT meta analysis ) AND LA English
**Cochrane**
((TI “pain” OR TX “pain assessment” OR TX “pain scale” OR TX “pain scales” OR TX “pain measurement” OR TX “pain measurements” OR TX “pain assessment tool” OR TX “pain assessment tools” OR TX “pain instrument” OR TX “pain instruments” OR TX “pain tool” OR TX “pain tools” OR TX “pain measure” OR TX “pain measures” OR TX “pain questionnaire” OR TX “pain questionnaires” OR TX “pain scale” OR TX “pain scales”) AND (TX “validity” OR TX “reliability” OR TX “psychometric” OR TX “valid” OR TX “reliable” OR TX “sensibility” OR TX “sensitivity” OR TX “accuracy” OR TX “responsiveness” OR TX “predictive value” OR TX “specificity” OR TX “simplicity”) AND (TX “hospitalization” OR TX “hospitalisation” AND TX “hospital” OR TX “inpatients” OR TX “inpatient”))

### 2.1 Screening Procedure

To ensure reliability, the screening procedure (i.e., title and abstract screening followed by full‐text screening) for including or excluding documents was performed independently by couples of researchers using the Rayyan web application (Ouzzani et al. 2016). In cases of conflict, consensus was reached through discussion, a third researcher being consulted if necessary. Before initiating the screening procedure, the researchers individually conducted a pilot exercise on the same set of 30 reviews to calibrate and test the assessment form.

### 2.2 Quality Appraisal

Quality appraisal was conducted using the JBI checklist for systematic reviews (Aromataris et al. 2015). Two researchers independently assessed all quality aspects, resolving discrepancies through consultation with a third researcher.

### 2.3 Data Extraction

Data extraction was performed independently by two researchers using a coding framework developed by the entire team to extract the following information: authors, publication year, title, quality appraisal tool used, timeframe of the included articles, searched bibliographic databases, number of included studies, population, sample size, setting, scale name, scale user (e.g., patient self‐report, caregiver, healthcare professional) and psychometric properties of the scale. To ensure consistent coding, three reviews were first analysed by seven researchers, followed by independent coding of all studies by two researchers, who then discussed and resolved discrepancies to achieve full agreement.

### 2.4 Data Analysis/Synthesis

For each scale, data extracted within the above‐described framework were summarised narratively. The summary of psychometric properties was guided by appropriate quality appraisal tools (e.g., the COSMIN checklist) (Prinsen et al. 2018) used in the reviews to evaluate these properties, ensuring a comprehensive and systematic integration of findings. For each psychometric property, the criteria for what constitutes good measurement properties were evaluated as sufficient, insufficient, indeterminate or inconsistent. Additionally, the risk of bias was assessed using standards to evaluate the methodological quality of studies assessing each property (i.e., very good, adequate, doubtful, inadequate), and the results were reported. Final evidence on the psychometric properties of the scales, if reported in the literature reviews, was summarised using the Grading of Recommendations Assessment, Development and Evaluation (GRADE) framework. The GRADE framework graded the overall quality of evidence as ‘high’, ‘moderate,’ ‘low’ or ‘very low.’ The COSMIN checklist (Prinsen et al. 2018) was used as the standard; however, in cases where some reviews used a different quality appraisal tool, the evaluations were mapped according to the terms used in COSMIN. For example, if another quality appraisal tool assessed the risk of bias as high, moderate, or low quality, these were mapped to the very good, adequate, and inadequate category, respectively. Additionally, a matrix of evidence for the entire overview was prepared and used to calculate the ‘corrected covered area’ (CCA) to quantify the degree of overlap between all the reviews included (Pieper et al. 2014).

## 3 Results

### 3.1 General Results

**FIGURE 1:**
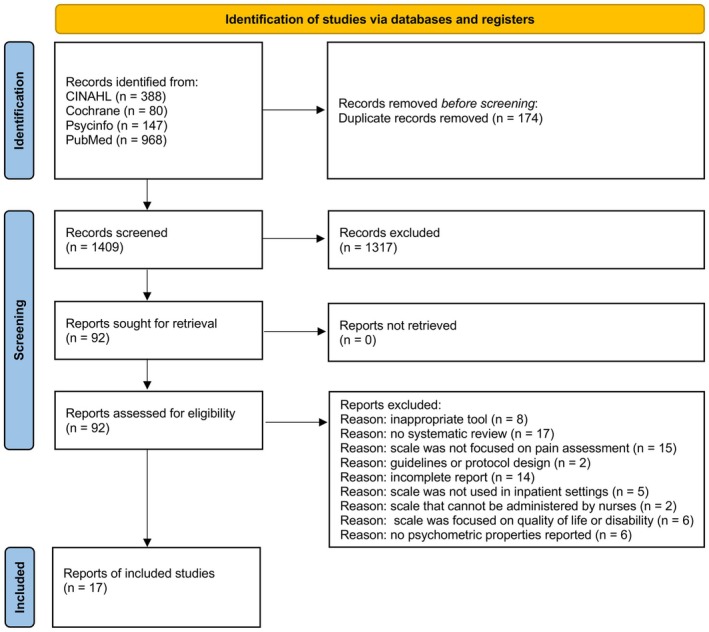
PRISMA 2020 study flow diagram. [Colour figure can be viewed at wileyonlinelibrary.com]

After excluding duplicates and articles not pertinent to the aim of the umbrella review, the full texts of 92 articles were screened and, ultimately, 17 records were finally included (Figure 1).

Appendix S2 and Table 2 report a summary of the main study results. The included reviews, published between 2014 and 2023, identified a total of 41 scales: 17 based on patient self‐reporting, 23 on observation by healthcare professionals or caregivers, and one on both self‐reporting and external observation. Scales were applied to different populations (e.g., paediatric or adults) or specific medical conditions (e.g., cancer or stroke). The most commonly used quality appraisal tool was the COSMIN checklist (*n* = 13; 76.5%), followed by the Quality Assessment of Diagnostic Accuracy Studies 2 (QUADAS‐2) (*n* = 2; 11.7%), a modified version of the Cochrane Collaboration's tool (*n* = 1; 5.9%), and the Quality Appraisal for Clinical Measurement Research Reports Evaluation (QACMRRE) (*n* = 1; 5.9%).

**TABLE 2:** Summary of reviewed studies, including population, sample size and scale details.

Authors	Databases used in the review	Time frame of the articles included in the review	Number of studies considered in the review according to our aim	Population	Sample size	Setting	Scale name	Brief description of the scale	Reporting style	Quality appraisal tool
Caravau et al. (2020)	PubMed, ScienceDirect, Web of Science, PEdro, Scielo, Scopus, and Academic Search Complete (EBSCO host) databases	2003	1	Individuals with cerebral palsy.	69	Clinical study	Verbal Rating Scale, 16‐point version (VRS‐16)	The VRS‐16 measures pain intensity using 16 verbal descriptors, from 0 (no pain) to 15 (unbearable pain). Higher scores indicate greater pain intensity	Self‐report	COSMIN checklist
Caravau et al. (2020)	PubMed, ScienceDirect, Web of Science, PEdro, Scielo, Scopus, and Academic Search Complete (EBSCO host) databases	2003	1	Individuals with cerebral palsy	69	Clinical study	21‐Point Numeric Rating Scale (NRS‐21)	The NRS‐21 measures pain intensity on a scale from 0 (no pain) to 20 (maximum pain). Higher scores indicate greater pain intensity	Self‐report	COSMIN checklist
Caravau et al. (2020)	PubMed, ScienceDirect, Web of Science, PEdro, Scielo, Scopus, and Academic Search Complete (EBSCO host) databases	2003	1	Individuals with cerebral palsy.	69	Clinical study	5‐Point Verbal Rating Scale (VRS‐5)	The VRS‐5 assesses pain intensity using 5 verbal descriptors, from 0 (no pain) to 4 (very severe pain). Higher scores indicate greater pain intensity	Self‐report	COSMIN checklist
Caravau et al. (2020)	PubMed, ScienceDirect, Web of Science, PEdro, Scielo, Scopus, and Academic Search Complete (EBSCO host) databases	2003	1	Individuals with cerebral palsy	69	Clinical study	7‐Point Faces Scales (FPS‐7)	The FPS‐7 assesses pain intensity using seven faces with emotional expressions, from 0 (no pain) to 6 (extreme pain). Higher scores indicate greater pain intensity	Self‐report	COSMIN checklist
Liossi et al. (2021)	Cochrane Library and PROSPERO were searched first, followed by EMBASE, CINAHL, MEDLINE (both via Ebsco), PsycINFO, the Web of Science Core Collection, the ProQuest Dissertations & Theses Database, Evidence Search, and OpenGrey	2014	1	Cancer	100	Cancer center (a hospital and a hospice)	Breakthrough Pain Assessment Tool (BAT)	The BAT is used to assess the intensity and characteristics of intermittent and sudden pain that occurs despite ongoing treatment. It includes 14 questions with nine addressing the pain itself and five focusing on its treatment. The BAT is moderately comprehensible for patients and clinicians, easy to administer and score, with a medium length and a completion time of 5–10 min	Self‐report	COSMIN checklist
Abahussin et al. (2019)	Medline (Ovid from 1996), Embase (Ovid from 1996), and the CINAHL (EBSCO from 1981)	1996–2011	8	Adult cancer patients	1223	Inpatient and outpatient	Brief Pain Inventory (BPI)	The BPI is a tool used to assess pain intensity and its impact on daily activities. It includes 15 questions about pain location, intensity and interference with life activities	Self‐report	COSMIN checklist
Smith et al. (2023)	MEDLINE, Embase, and PsycInfo	2018	1	Children with cerebral palsy unable to self‐report	167	Hospital				COSMIN checklist
Abahussin et al. (2019)	Medline (Ovid from 1996), Embase (Ovid from 1996), and the CINAHL (EBSCO from 1981)	2002–2008	3	Adult cancer patients	575	Inpatient and outpatient	BPI‐Short Form (BPI‐SF)	The BPI‐SF is a shortened version of the BPI, used to assess pain intensity and its impact on daily functions. It includes 9 questions about pain location, intensity and interference with activities	Self‐report	COSMIN checklist
Abahussin et al. (2019)	Medline (Ovid from 1996), Embase (Ovid from 1996), and the CINAHL (EBSCO from 1981)	2010	1	Adult cancer patients	262	Inpatient and outpatient	Cancer Pain Inventory (CPI)	The CPI is a tool designed to assess pain intensity and characteristics in cancer patients. It includes 10 questions about pain intensity, interference with functioning, emotional responses to pain (e.g., catastrophizing), the social aspect of pain and the use of pain medication. It was moderately comprehensible for patients and clinicians, with administration and scoring rated as moderately easy	Self‐report	COSMIN checklist
Dorfman et al. (2014)	MEDLINE, CINAHL, EMBASE, Web of Science (WOS), BIOSIS Previews, Cochrane Central Register of Controlled Trials, Cochrane Database of Systematic Reviews, Database of Abstracts of Review and Effects (DARE), Scopus, and Proquest Dissertations	2004	1	Mechanically ventilated Paediatric Intensive Care Unit (PICU) patients	69	PICU	Cardiac Analgesic Assessment Scale (CAAS)	The CAAS is based on four physiological and one behavioural variables. The highest aggregate score is 8, pain is assumed with a score of ≥ 4	Observation	COSMIN checklist
Dorfman et al. (2014)	MEDLINE, CINAHL, EMBASE, Web of Science (WOS), BIOSIS Previews, Cochrane Central Register of Controlled Trials, Cochrane Database of Systematic Reviews, Database of Abstracts of Review and Effects (DARE), Scopus, and Proquest Dissertations	2000–2001	1	Mechanically ventilated Paediatric Intensive Care Unit (PICU) patients	204	PICU	COMFORT	The COMFORT Scale measures the level of pain, distress and sedation. The total score ranges from 8 to 40. Lower scores indicate adequate sedation or pain control. The Comfort Scale is user‐friendly, requiring 2–3 min for administration and about 2 h of training for healthcare professionals. It has good interpretability, avoiding ceiling or floor effects and accommodating varying distress levels	Observation	COSMIN checklist
Maaskant et al. (2016)	Central, CINAHL, Embase, Medline, PsycInfo, and Web of Science	1998–2013	8	Children from birth until 18 years, including premature neonates	565	Highly specialised paediatric intensive care units (PICUs); Neonatal Intensive Care Units (NICUs); operation room, a specialised Burn Hospital and on a maternity ward				COSMIN checklist
Maaskant et al. (2016)	Central, CINAHL, Embase, Medline, PsycInfo, and Web of Science	2007	1	Children from birth until 18 years, including premature neonates	57	Highly specialised paediatric intensive care units (PICUs); Neonatal Intensive Care Units (NICUs); operation room, a specialised Burn Hospital and on a maternity ward	COMFORT‐without blood pressure	This tool is a variant of the COMFORT Scale which omits blood pressure measurement. The total score ranges from 7 to 35. Lower scores indicate adequate sedation or pain control	Observation	COSMIN checklist
Dorfman et al. (2014)	MEDLINE, CINAHL, EMBASE, Web of Science (WOS), BIOSIS Previews, Cochrane Central Register of Controlled Trials, Cochrane Database of Systematic Reviews, Database of Abstracts of Review and Effects (DARE), Scopus, and Proquest Dissertations.	2000–2011	3	Mechanically ventilated Paediatric Intensive Care Unit (PICU) patients	664	PICU	COMFORT‐Behavioural (COMFORT‐B) scale	The COMFORT Scale—B is a tool used to assess the level of pain, distress and sedation in patients. It focuses on observable behaviours, such as facial expressions, body movements and responses to stimuli, to evaluate the patient's well‐being. The total score ranges from 8 to 40, with lower scores indicating adequate pain and sedation control. The Comfort‐B scale is easy to integrate into nursing care, with a 2‐h training time	Observation	COSMIN checklist
Maaskant et al. (2016)	Central, CINAHL, Embase, Medline, PsycInfo, and Web of Science	2000–2014	9	Children from birth until 18 years, including premature neonates	1454	Highly specialised paediatric intensive care units (PICUs); Neonatal Intensive Care Units (NICUs); operation room, a specialised Burn Hospital and on a maternity ward				COSMIN checklist
Maaskant et al. (2016)	Central, CINAHL, Embase, Medline, PsycInfo, and Web of Science	2009	1	Children from birth until 18 years, including premature neonates	174	Highly specialised paediatric intensive care units (PICUs); Neonatal Intensive Care Units (NICUs); operation room, a specialised Burn Hospital and on a maternity ward	COMFORT‐neo	The COMFORT‐neo is a scale used to assess pain, distress and sedation in neonates and young children. It measures behaviour and physiological responses, such as heart rate, blood pressure and respiration, to determine the level of pain or discomfort. The total score ranges from 8 to 40, with lower scores indicating adequate pain and sedation control	Observation	COSMIN checklist
Zhai et al. (2020)	MEDINE, CINAHL, ProQuest, EMBASE, Cochrane Library, CNKI, Wanfang, COVIP, and CBM.	2007–2019	25	Intensive Care Unit (ICU) patients	3220	ICU, hospital	Critical‐Care Pain Observation Tool (CPOT)	The CPOT is used to evaluate pain in intubated or sedated patients by observing facial expressions, muscle tension and body movements, along with ventilator compliance for intubated patients or vocal expressions of pain for non‐intubated patients. The assigned scores may range from 0 to 8 points.	Self‐report	QUADAS‐2
Smith et al. (2023)	MEDLINE, Embase, and PsycInfo.	2010–2014	2	Elderly (most of them affected by dementia) unable to self‐report	468	Long‐term care setting, hospital	Doloplus‐2	The Doloplus‐2 scale assesses pain in elderly individuals with verbal communication disorders using 10 items scored 0–3: five somatic, two psychomotor and three psychosocial	Observation	COSMIN checklist
Liossi et al. (2021)	Cochrane Library and PROSPERO were searched first, followed by EMBASE, CINAHL, MEDLINE (both via Ebsco), PsycINFO, the Web of Science Core Collection, the ProQuest Dissertations & Theses Database, Evidence Search, and OpenGrey.	2019	1	Cancer	170	Hospitals, hospice, medical centres, cancer institute	BAT‐dutch version	The Dutch version of the BAT retains the original BAT structure, comprising 14 questions: nine assessing pain characteristics and five evaluating pain treatment efficacy	Self‐report	COSMIN checklist
Crellin et al. (2015)	MEDLINE, CINAHL, Embase, PsycINFO, Cochrane Database of Systematic reviews, Cochrane Controlled Trials, and Google Scholar.	1997–2013	26	Infants (from birth to 1 year) and children (from 1 to 18 years)	2240	Inpatient and outpatient center	Face, Legs, Activity, Cry and Consolability (FLACC) scale	The FLACC scale assesses pain in nonverbal individuals by scoring five behaviours (face, legs, activity, cry, consolability) from 0 to 2, with a total score of 0–10 indicating pain severity. The FLACC scale is simple, quick and does not assess physiological variables, but its feasibility remains uncertain. While limited to acute pain assessment, it has high clinical usefulness	Observation	COSMIN checklist
Dorfman et al. (2014)	MEDLINE, CINAHL, EMBASE, Web of Science (WOS), BIOSIS Previews, Cochrane Central Register of Controlled Trials, Cochrane Database of Systematic Reviews, Database of Abstracts of Review and Effects (DARE), Scopus, and Proquest Dissertations	2003–2009	2	Mechanically ventilated Paediatric Intensive Care Unit (PICU) patients	51	PICU				COSMIN checklist
Baamer et al. (2022)	MEDLINE, Embase, CINAHL, and PsycINFO	2008–2015	6	Hospitalised surgical adult patients	929	Hospital	6‐Point Faces Pain Scale (FPS‐6)	The FPS‐6 is a 7‐point horizontal scale featuring images of facial expressions to represent varying pain levels. Patients choose the face that best reflects their current pain, with 0 indicating “no pain” and 6 representing “the worst possible pain”	Self‐report	Mod. NOS and COSMIN
Caravau et al. (2020)	PubMed, ScienceDirect, Web of Science, PEdro, Scielo, Scopus, and Academic Search Complete (EBSCO host) databases.	2003	1	Individuals with cerebral palsy	69	Clinical study				COSMIN checklist
de Vries et al. (2016)	PubMed (Medline), PsychINFO, CINAHL, EMBASE, Web of Science, and Cochrane.	2007	1	Stroke patients with aphasia	127	Rehabilitation setting				COSMIN checklist
Edwards et al. (2020)	Medline (Ovid), Embase (Ovid), CINAHL (EBSCO), and PsychInfo (EBSCO).	2007–2010	2	Adult stroke survivors at any stage of recovery	187	Rehabilitation setting				QUADAS‐2
Liossi et al. (2021)	Cochrane Library and PROSPERO were searched first, followed by EMBASE, CINAHL, MEDLINE (both via Ebsco), PsycINFO, the Web of Science Core Collection, the ProQuest Dissertations & Theses Database, Evidence Search, and OpenGrey.	2017	1	Cancer	120	Pain management clinic	BAT‐Korean version (BAT‐K)	Korean Version of the Breakthrough Pain Assessment Tool (K‐BAT) retains the original BAT's structure, comprising 14 questions: nine addressing pain characteristics and five evaluating the effectiveness of pain treatment	Self‐report	COSMIN checklist
Liossi et al. (2021)	Cochrane Library and PROSPERO were searched first, followed by EMBASE, CINAHL, MEDLINE (both via Ebsco), PsycINFO, the Web of Science Core Collection, the ProQuest Dissertations & Theses Database, Evidence Search, and OpenGrey.	2016	1	Chronic pain (cancer or non cancer)	120	Hospital	Italian Questionnaire for Breakthrough Pain (IQ‐BTP)	The Italian Questionnaire for BTcP diagnosis (IQ‐BTP) is an 11‐item tool designed to identify potential breakthrough cancer pain and classify it into three likelihood categories: high, intermediate and low. The IQ‐BTP was moderately easy to use, with moderate comprehensibility and a completion time of about 5 min	Observation	COSMIN checklist
Mulvey et al. (2017)	MEDLINE, EMBASE, and CINAHL.	2003–2015	4	Cancer patients with a clinical diagnosis of neuropathic or not neuropathic pain	764	Inpatient and outpatient center	Leeds Assessment of Neuropathic Symptoms and Signs (LANSS)	The LANSS is a scale that combines a symptom questionnaire and sensory examination to assess neuropathic pain. A score above a threshold suggests neuropathic pain	Observation	Mod. Cochrane Coll.
Abahussin et al. (2019)	Medline (Ovid from 1996), Embase (Ovid from 1996), and the CINAHL (EBSCO from 1981)	2002	1	Adult cancer patients	114	Inpatient and outpatient	McGill Pain Questionnaire	The McGill Pain Questionnaire assesses pain through descriptive words, a pain intensity scale and a body diagram for pain location, providing a detailed understanding of sensory, affective and evaluative pain aspects	Self‐report	COSMIN checklist
Crellin et al. (2018)	MEDLINE, EMBASE, Cochrane Database of Systematic Reviews, Cochrane Controlled Trials, CINAHL, and PsycINFO	1995–2015	12	Infants and children aged 2 months to 18 years	854	Inpatient and outpatient center	Modified Behavioural Pain Scale (MBPS)	The MBPS is a tool used to assess pain in intubated patients, particularly in intensive care units. It is based on the observation of specific behaviours, such as limb movement, facial expression and response to the ventilator, to determine the level of pain. The total score helps monitor and manage pain in these patients	Observation	COSMIN checklist
Smith et al. (2023)	MEDLINE, Embase, and PsycInfo	2018	1	Children with cerebral palsy unable to self‐report	167	Hospital	Modified Brief Pain Inventory (mBPI)	The BPI was designed to measure pain intensity and its impact on activities of daily living, with its modified version later adapted for adults with cerebral palsy and youth with neuromuscular diseases	Observation	COSMIN checklist
Dorfman et al. (2014)	MEDLINE, CINAHL, EMBASE, Web of Science (WOS), BIOSIS Previews, Cochrane Central Register of Controlled Trials, Cochrane Database of Systematic Reviews, Database of Abstracts of Review and Effects (DARE), Scopus, and Proquest Dissertations	2009	1	Mechanically ventilated Paediatric Intensive Care Unit (PICU) patients	40	PICU	Modified version of FLACC (mFLACC)	The Modified FLACC scale assesses pain in children by evaluating five behaviours—facial expression, leg movement, bodily activity, cry or verbalization (adjusted for ventilated or non‐ventilated patients) and consolability	Observation	COSMIN checklist
Dorfman et al. (2014)	MEDLINE, CINAHL, EMBASE, Web of Science (WOS), BIOSIS Previews, Cochrane Central Register of Controlled Trials, Cochrane Database of Systematic Reviews, Database of Abstracts of Review and Effects (DARE), Scopus, and Proquest Dissertations	2007	2	Mechanically ventilated Paediatric Intensive Care Unit (PICU) patients	63	PICU	Multidimensional Assessment of Pain Scale (MAPS)	The MAPS evaluates pain by assessing vital signs (heart rate and/or blood pressure), breathing pattern, facial expressions, body movements and state of arousal	Observation	COSMIN checklist
Edwards et al. (2020)	Medline (Ovid), Embase (Ovid), CINAHL (EBSCO), and PsycInfo (EBSCO).	2011	1	Adult stroke survivors at any stage of recovery	19	Rehabilitation setting	Neuropathic Pain Diagnostic Questionnaire (DN4)	The DN4 questionnaire combines pain descriptors and a sensory exam, with a score of 4 or higher indicating predominantly neuropathic origin	Self‐report	QUADAS‐2
Vink et al. 2017	CENTRAL, CINAHL, Embase, PsycInfo, and Web of Science	2009–2015	5	Adult patients (> 18 years) with disorders of consciousness due to acquired brain injury	166	Semi acute and long‐term settings	Nociception Coma Scale (NCS)	The NCS evaluates nociceptive behaviour by assessing motor response, verbal response, facial expression and visual response	Observation	COSMIN checklist
Vink et al. 2017	CENTRAL, CINAHL, Embase, PsycInfo, and Web of Science	2012–2015	5	Adult patients (> 18 years) with disorders of consciousness due to acquired brain injury	182	Semi acute and long‐term settings	Nociception Coma Scale‐Revised (NCR‐r)	The NCR‐R is a scale used to assess pain response in comatose patients. It focuses on physiological and behavioural indicators to identify pain perception in unconscious patients. The final score helps monitor the level of nociception and guide pain management.	Observation	COSMIN checklist
Baamer et al. (2022)	MEDLINE, Embase, CINAHL, PsycINFO	2003–2016	13	Hospitalised adult patients	7096	Hospital	Numeric Pain Rating Scale (NPRS)	The Numeric Pain Rating Scale (NPRS) is a tool used to measure pain intensity. The patient rates their pain on a numerical scale from 0 to 10, where 0 represents no pain and 10 represents the worst pain imaginable. The NPRS is easy to implement in clinical practice	Self‐report	Mod. NOS and COSMIN
Chiarotto et al. (2019)	MEDLINE, EMBASE, CINAHL, PsycINFO, SportDiscus, and Google Scholar.	1991–2015	11	Patients with nonspecific low back pain	2709	Inpatient and outpatient center				COSMIN checklist
Edwards et al. (2020)	Medline (Ovid), Embase (Ovid), CINAHL (EBSCO), and PsycInfo (EBSCO)	2013	1	Adult stroke survivors at any stage of recovery	388	Acute stroke unit setting				QUADAS‐2
Modarresi et al. (2021)	CINAHL, Embase, PsycInfo, and MedLine.	1997–2020	22	Participants with neck pain of mechanical, traumatic or nonspecific origin, with or without radiculopathy	11,788	Inpatient and outpatient center				QACMRRE
Caravau et al. (2020)	PubMed, ScienceDirect, Web of Science, PEdro, Scielo, Scopus, and Academic Search Complete (EBSCO host) databases.	2003	1	Individuals with cerebral palsy	45	Clinical study				COSMIN checklist
Caravau et al. (2020)	PubMed, ScienceDirect, Web of Science, PEdro, Scielo, Scopus, and Academic Search Complete (EBSCO host) databases.	2013	1	Children with severe cerebral palsy and other neurological conditions	100	Inpatient	Paediatric Pain Profile (PPP)	The PPP is a tool based on the observation of behaviours and physiological signs. The overall score helps monitor and manage pain in children who cannot verbally express themselves.	Observation	COSMIN checklist
Smith et al. (2023)	MEDLINE, Embase, and PsycInfo	2004–2007	2	Children aged 1 to 18 years unable to self‐report	169	Hospice, tertiary referral hospital, health service respite care centre				COSMIN checklist
Edwards et al. (2020)	Medline (Ovid), Embase (Ovid), CINAHL (EBSCO), and PsycInfo (EBSCO).	2018	1	Adult stroke survivors at any stage of recovery	36	Acute stroke unit setting	Pain Assessment Scale for Seniors with Severe Dementia‐II (PACSLAC‐II)	The PACSLAC‐II is a tool designed to assess pain in elderly patients with severe dementia who cannot communicate verbally. It is based on 60 observable behaviours and signs, such as facial expressions, posture and motor activity, to detect the presence of pain	Observation	QUADAS‐2
Caravau et al. (2020)	PubMed, ScienceDirect, Web of Science, PEdro, Scielo, Scopus, and Academic Search Complete (EBSCO host) databases	2001–2010	2	Patients with severe cerebral palsy (children, adolescents and adults)	408	Hospital, rehabilitation centres	Pain Evaluation Scale (PES)	The PES assesses pain intensity and characteristics through questions and behavioural observations. The final score ranges from 0 (no pain) to 10 (maximum pain)	Observation	COSMIN checklist
Chiarotto et al. (2019)	MEDLINE, EMBASE, CINAHL, PsycINFO, SportDiscus, and Google Scholar	2004–2015	4	Patients with nonspecific low back pain	621	Inpatient and outpatient center	Pain Severity sub‐scale of the Brief Pain Inventory (BPI‐PS)	The BPI‐PS is a component of the Brief Pain Inventory used to assess pain intensity. It consists of a series of questions asking the patient to rate the intensity of their pain at different times of the day. The total score ranges from 0 (no pain) to 10 (maximum pain)	Observation	COSMIN checklist
Mulvey et al. (2017)	MEDLINE, EMBASE, and CINAHL	2013–2015	2	Cancer patients with a clinical diagnosis of neuropathic or not neuropathic pain	1409	Inpatient and outpatient center	painDETECT screening tool (PDQ)	The PDQ is a tool that identifies neuropathic pain through 9 questions. The score ranges from 0 to 38, with higher scores indicating a higher likelihood of neuropathic pain	Observation	Mod. Cochrane Coll.
Caravau et al. (2020)	PubMed, ScienceDirect, Web of Science, PEdro, Scielo, Scopus, and Academic Search Complete (EBSCO host) databases	2014–2017	2	Children/adolescents with cerebral palsy (age 2–17 years) who experience limb spasticity‐related pain	170	Inpatient	Questionnaire on Pain caused by Spasticity (QPS)	The QPS assesses spasticity‐related pain with 15 questions. The total score ranges from 0 to 60, with higher scores indicating a greater impact on quality of life	Mixed (Self‐report + observation)	COSMIN checklist
Liossi et al. (2021)	Cochrane Library and PROSPERO were searched first, followed by EMBASE, CINAHL, MEDLINE (both via Ebsco), PsycINFO, the Web of Science Core Collection, the ProQuest Dissertations & Theses Database, Evidence Search, and OpenGrey	2012	1	Cancer	229	Palliative care centres	Questionnaire for Intense Episodic Pain (QUEDI)	The QUEDI assesses intense episodic pain with 10–12 questions. The total score measures the severity and impact of these episodes on the patient's daily life. It was moderately comprehensible for patients and clinicians, with a completion time of about 10 min	Observation	COSMIN checklist
Caravau et al. (2020)	PubMed, ScienceDirect, Web of Science, PEdro, Scielo, Scopus, and Academic Search Complete (EBSCO host) databases	2015–2019	2	Young people and adults with spastic cerebral palsy	75	Inpatient and outpatient	Revised‐FLACC	The revised FLACC scale is an adapted version of the FLACC and assesses pain in nonverbal individuals by scoring five behaviours (face, legs, activity, cry and consolability) from 0 to 2. It includes updated descriptors for more accurate pain evaluation, with a total score ranging from 0 to 10	Observation	COSMIN checklist
Baamer et al. (2022)	MEDLINE, Embase, CINAHL, and PsycINFO	1989–2011	7	Hospitalised adult patients	5167	Hospital	Verbal Rating Scale (VRS)	The VRS assesses pain intensity with verbal categories. If it is necessary to quantify the pain numerically, usually 0, 1, 2 or 3 points are assigned to the expressions mentioned above, respectively	Self‐report	Mod. NOS and COSMIN
de Vries et al. (2016)	PubMed (Medline), PsycINFO, CINAHL, EMBASE, Web of Science, and Cochrane	2007	1	Stroke patients with aphasia	127	Rehabilitation setting				COSMIN checklist
Baamer et al. (2022)	MEDLINE, Embase, CINAHL, and PsycINFO	1982–2018	20	Hospitalised adult patients	6679	Hospital	Visual Analogue Scale (VAS)	The VAS is a tool used to measure pain intensity. It consists of a continuous line, usually 10 cm long, where 0 indicates no pain and 10 cm represents the worst pain imaginable. VAS is easy to implement and provides clear, consistent scores	Self‐report	Mod. NOS and COSMIN
Chiarotto et al. (2019)	MEDLINE, EMBASE, CINAHL, PsycINFO, SportDiscus, and Google Scholar	1989–2015	9	Patients with nonspecific low back pain	1417	Inpatient and outpatient center				COSMIN checklist
Dabbagh et al. (2022)	CINAHL, MEDLINE (through Ovid, from 1946), Embase, and Scopus	2013–2018	2	Patients with ulnar nerve entrapment at the elbow	116	Surgical inpatient unit				COSMIN checklist
Edwards et al. (2020)	Medline (Ovid), Embase (Ovid), CINAHL (EBSCO), and PsycInfo (EBSCO).	1999–2017	4	Adult stroke survivors at any stage of recovery	441	Acute stroke unit setting				QUADAS‐2
Modarresi et al. (2021)	CINAHL, Embase, PsycInfo, and MedLine	2010–2020	7	Participants with neck pain of mechanical, traumatic, or nonspecific origin, with or without radiculopathy	1741	Hospital, clinic				QACMRRE
de Vries et al. (2016)	PubMed (Medline), PsycINFO, CINAHL, EMBASE, Web of Science, and Cochrane	1999 and 2007	4	Stroke patients with aphasia	394	Inpatient and outpatient center				COSMIN checklist
Liossi et al. (2021)	Cochrane Library. PROSPERO, EMBASE, CINAHL, MEDLINE, PsycINFO, Web of Science Core Collection, ProQuest Dissertations, Evidence Search, and OpenGrey	2013–2015	1	Cancer	135	Cancer center (a hospital and a hospice)	Webber's Breakthrough Cancer Pain Algorithm	The Webber's Breakthrough Cancer Pain Algorithm guides the management of breakthrough cancer pain, recommending fast‐acting pain medications, such as opioids, based on pain assessment. The algorithm was easy to administer but difficult for patients and clinicians to understand	Observation	COSMIN checklist

Abbreviations: COSMIN checklist, COnsensus‐based Standards for the selection of health Measurement Instruments; Mod. Cochrane Coll., modified version of the Cochrane Collaboration's tool; Mod. NOS, modified version of the Newcastle Ottawa Scale; QACMRRE, Quality Appraisal for Clinical Measurement Research Reports Evaluation; QUADAS‐2, Quality Assessment of Diagnostic Accuracy Studies 2 tool.

### 3.2 Psychometric Characteristics

#### 3.2.1 Breakthrough Pain Assessment Tool (BAT)

The BAT was evaluated in one literature review published in 2021, which included one article about this scale published in 2014 (Liossi et al. 2021). Only one article assessed the BAT, with a sample size of 100 patients from two hospital cancer centres. As for the BAT validity, the content, structural, and construct validity were shown to be sufficient in the study, with varying quality levels; the evidence for content validity was of a low level. The internal reliability and test–retest reliability were demonstrated to be sufficient in one study of very good and doubtful quality, respectively. The BAT was found to be responsive to clinical interventions, and the evidence for its responsiveness was high.

#### 3.2.2 BAT‐Dutch Version (BAT‐DL)

The BAT‐DL was evaluated in one literature review published in 2021, which included one article published in 2019 (Liossi et al. 2021). The tool was included in only one study conducted in three hospitals, one hospice, two medical centres, and one cancer institute. The study included 170 cancer patients.

As for the BAT‐DL validity, construct validity was shown to be sufficient in one study of very good quality. Structural validity showed indeterminate evidence in one study of very good quality. Content validity was shown to be indeterminate in one study of doubtful quality, providing low evidence of its validity. The internal reliability was found to be indeterminate in one study of very good quality, while test–retest reliability showed sufficient evidence in one study of doubtful quality, demonstrating low evidence of reliability. Regarding responsiveness, very low evidence was reported. Although the study rated responsiveness as sufficient, its methodological quality was inadequate.

#### 3.2.3 BAT‐Korean Version (BAT‐K)

The BAT‐K was evaluated in one literature review published in 2021, which included one article published in 2017 (Liossi et al. 2021). The tool was assessed in only one study conducted in a pain management clinic with a sample of 120 cancer patients. As for the BAT‐K validity, structural validity was shown to be indeterminate in one study of adequate quality. Content validity was shown to be indeterminate in one study of doubtful quality, providing low evidence of it. Internal reliability was shown to be sufficient in one study of very good quality. Test–retest reliability also showed sufficient evidence in one study of doubtful quality, demonstrating low evidence of its reliability. Responsiveness was shown to be indeterminate in one study of adequate quality.

#### 3.2.4 Brief Pain Inventory (BPI)

The BPI was evaluated in two literature reviews, one published in 2019 (Abahussin et al. 2019) and the other in 2023 (Smith et al. 2023). The time frame of the articles included in the two reviews was from 1996 to 2011. A total of 8 studies were considered in the 2019 review, and one article was included in the 2023 review. The studies evaluated a population of adult cancer patients, with 1223 patients in the 2019 review and 27 patients in the 2023 review. The settings were inpatient and outpatient for the 2019 review, while the 2023 study was conducted at a cancer centre.

As for BPI validity, construct validity was reported to be sufficient in six studies, although the methodological quality of these studies was inadequate or doubtful. Structural validity was rated as indeterminate or insufficient in eight studies, with only one study being of very good methodological quality. Internal reliability was found to be sufficient in all eight studies, three of which were of adequate or very good methodological quality. Inter‐rater reliability and test–retest reliability were reported as insufficient in one study of very good quality and one of inadequate quality, respectively, demonstrating low evidence for the scale's reliability.

#### 3.2.5 Modified BPI (mBPI)

The mBPI was evaluated in a 2023 systematic review (Smith et al. 2023), which included one 2018 study of 167 children with cerebral palsy (unable to self‐report) in a hospital setting. Construct validity was assessed in one study of very good quality, which provided sufficient evidence to support it. Internal reliability was evaluated in one study of doubtful quality, which provided sufficient evidence but demonstrated a low level of internal reliability due to a lack of reported structural validity.

#### 3.2.6 Pain Severity Subscale of the BPI‐Pain Severity Subscale (BPI‐PS)

The BPI‐PS was evaluated in a literature review published in 2019 (Chiarotto et al. 2019), which covered articles from 2004 to 2015. The review assessed a total of four articles, involving over 600 adults with nonspecific low back pain. Moderate evidence for inconsistent construct validity of the BPI‐PS was demonstrated in two studies. One study of adequate quality found sufficient results for structural validity. Low evidence for content validity was shown by one study. As for reliability, two studies of adequate quality showed sufficient internal reliability. Two studies of inadequate quality showed inconsistent results for responsiveness.

#### 3.2.7 BPI‐Short Form (BPI‐SF)

The BPI‐SF was evaluated in a systematic review published in 2019 (Abahussin et al. 2019). The review considered articles published between 2002 and 2008, with a total of 3 studies assessing the BPI‐SF. The studies included 575 adult cancer patients in inpatient and outpatient settings.

For BPI‐SF validity, construct validity was rated as sufficient in three studies of inadequate or doubtful methodological quality. Structural validity was rated as sufficient in two studies of doubtful methodological quality and indeterminate in one study of adequate quality. Internal reliability was demonstrated as sufficient in two studies; one was of adequate quality and indeterminate in one study of doubtful quality.

#### 3.2.8 Cancer Pain Inventory (CPI)

The CPI was evaluated in a 2019 literature review, which included one 2010 study of 262 adult cancer patients in inpatient and outpatient settings (Abahussin et al. 2019). As for CPI validity, the construct validity was rated as sufficient. The structural validity was assessed as indeterminate and the internal reliability was shown to be insufficient; the quality of the study was of doubtful quality for all these three properties.

#### 3.2.9 Cardiac Analgesic Assessment Scale (CAAS)

The CAAS was evaluated in a literature review published in 2014 (Dorfman et al. 2014). A single study, published in 2004, was considered for the CAAS in this review, involving 69 mechanically ventilated patients in a Paediatric Intensive Care Unit (PICU). Criterion validity was judged insufficient. Inter‐rater reliability and responsiveness of the CAAS were rated as sufficient; but the study was of doubtful methodological quality.

#### 3.2.10 COMFORT

The COMFORT was evaluated in two literature reviews, published in 2014 (Dorfman et al. 2014) and 2016 (Maaskant et al. 2016), which covered studies from 1998 to 2013. These reviews included a total of nine articles, encompassing over 700 neonates or paediatric patients who were assessed using this scale. The construct validity of the scale was rated as sufficient by three studies, two of doubtful quality, one of adequate quality, and as insufficient by one study of inadequate methodological quality. Internal consistency and inter‐rater reliability were supported as sufficient in five and three studies, respectively. The COMFORT's responsiveness was rated as sufficient in four studies, mostly of adequate or very good methodological quality.

#### 3.2.11 COMFORT‐Behavioural (COMFORT‐b)

The COMFORT‐b was evaluated in two literature reviews published in 2014 (Dorfman et al. 2014) and 2016 (Maaskant et al. 2016), which analysed studies from 2000 to 2014. These reviews included nine articles, involving over 1400 neonates or paediatric patients assessed using the scale. Construct validity was rated as sufficient in six studies, mostly of doubtful quality. In terms of criterion validity, one study of very good quality indicated that the scale has sufficient criterion validity. For internal reliability, four studies mostly of adequate methodological quality showed sufficient internal consistency. Sufficient inter‐rater reliability was also supported by five studies, four of which were of very good or adequate methodological quality. COMFORT‐b responsiveness was rated as sufficient in two studies of adequate and doubtful quality, respectively.

#### 3.2.12 COMFORT‐Neo (COMFORT‐n)

The COMFORT‐n was evaluated in a 2016 systematic review (Maaskant et al. 2016), which included one 2009 study of 174 paediatric patients (neonates to 18 years) in PICUs, neonatal intensive care units, operating rooms, burn hospitals, and maternity wards. Construct validity and internal consistency were rated as sufficient, although the study was of doubtful methodological quality. Inter‐rater reliability was reported as sufficient in a study of adequate quality. Responsiveness was rated as sufficient in a study of very good methodological quality.

#### 3.2.13 COMFORT‐Without Blood Pressure (COMFORT‐w)

In a 2016 systematic review (Maaskant et al. 2016), the COMFORT‐w was evaluated in one 2007 study of 57 children (premature neonates to 18 years) in paediatric and maternity facilities. This scale showed sufficient construct validity; however, the methodological quality of the study was inadequate, reducing the level of evidence of this validity. Internal consistency and responsiveness were rated as sufficient in a study of adequate quality. Inter‐rater reliability was rated as sufficient; however, the study was of doubtful methodological quality. The scale showed very good sensitivity (0.93) and acceptable specificity (0.80).

#### 3.2.14 Critical‐Care Pain Observation Tool (CPOT)

The CPOT was evaluated in a systematic review and meta‐analysis published in 2020 (Zhai et al. 2020), covering 25 studies conducted between 2007 and 2019. The CPOT was used in 3220 intensive care unit patients. The 25 studies of doubtful methodological quality due to a high risk of bias in terms of patient selection, flow, and timing, reported both suboptimal sensitivity (74.3%) and specificity (76.8%).

#### 3.2.15 Doloplus‐2

The Doloplus‐2 was considered in a 2023 review (Smith et al. 2023), in two articles published from 2010 to 2014. Four hundred and sixty‐eight elderly patients, most of them affected by dementia and unable to self‐report, were assessed with this scale in both long‐term care settings and hospitals. The content validity demonstrated sufficient evidence in a study of inadequate quality. Indeterminate evidence was reported for both structural and cross‐cultural validity in a study of inadequate quality, while construct validity demonstrated sufficient evidence in a study of adequate quality. As for reliability, internal consistency revealed indeterminate evidence in one study of very good quality, while inter‐rater reliability indicated sufficient evidence in two studies of adequate quality.

#### 3.2.16 Face, Legs, Activity, Cry and Consolability (FLACC)

The FLACC was considered in two reviews published in 2014 (Dorfman et al. 2014) and 2015 (Crellin et al. 2015), which included 28 articles from 1997 to 2013. A total of 2291 infants and children, both inpatients and outpatients, were evaluated using the FLACC. Content validity was supported by insufficient evidence. In contrast, construct validity was supported by sufficient evidence in 11 studies—two of which were conducted in mechanically ventilated patients—and by poor evidence in four additional studies. Structural validity was supported with sufficient evidence in four studies. Criterion validity showed sufficient evidence in five studies; two of these five studies were conducted in mechanically ventilated patients, while six studies provided poor evidence. Internal reliability was supported by sufficient evidence in four studies; one of these studies was conducted in mechanically ventilated patients, whereas two studies provided poor evidence. Inter‐rater reliability was demonstrated as sufficient in 14 studies; two of these 14 studies were conducted in mechanically ventilated patients, while three studies showed poor evidence. Responsiveness of the FLACC scale was indicated as poor in twelve studies, while eight studies provided sufficient evidence.

#### 3.2.17 Modified Version of FLACC (mFLACC)

The mFLACC was examined in a systematic review published in 2014 (Dorfman et al. 2014). This review included one article, published in 2009, that evaluated the scale in 40 mechanically ventilated patients in a PICU. In terms of construct validity, one study of doubtful quality demonstrated sufficient evidence. Criterion validity was also supported with sufficient evidence in a study of adequate quality. Regarding reliability, inter‐rater reliability was shown to have sufficient evidence based on findings from one study, which was evaluated as of adequate quality.

#### 3.2.18 Revised‐FLACC

The revised‐FLACC was analysed in a 2020 review (Caravau et al. 2020); two articles published between 2015 and 2019 considered this scale in 75 young people and adults with spastic cerebral palsy, in both inpatient and outpatient settings. Construct validity was found to be sufficient in one study of doubtful methodological quality, while criterion validity exhibited indeterminate evidence in one study of very good quality, providing low evidence for both properties. Internal reliability revealed sufficient evidence in one study of very good quality, while inter‐rater reliability showed insufficient and sufficient evidence in two studies of very good quality and doubtful quality, respectively.

#### 3.2.19 Italian Questionnaire for Breakthrough Pain (IQ‐BTP)

The IQ‐BTP was evaluated in a 2021 systematic review, which included one 2016 study of 120 patients with chronic pain (cancer or non‐cancer) in a hospital setting (Liossi et al. 2021). For the IQ‐BTP's validity, construct validity was demonstrated as sufficient in the study, with high‐quality evidence supporting this conclusion. However, evidence for structural validity was indeterminate and content validity was inconsistent in a study of inadequate quality, providing low evidence for content validity. Internal reliability was rated as sufficient, providing high evidence for this property.

#### 3.2.20 Leeds Assessment of Neuropathic Symptoms and Signs (LANSS)

The LANSS was investigated in a 2017 review, which included four articles published between 2003 and 2015 (Mulvey et al. 2017). A total of 764 cancer patients, with a clinical diagnosis of either neuropathic or non‐neuropathic pain, were assessed using the LANSS in both inpatient and outpatient settings. The LANSS demonstrated a sensitivity ranging from 86% to 29.5% and specificity from 100% to 91% in the four studies with a low risk of bias related to its administration and the reporting of its performance values. However, variation in the risk of bias was observed in clinician assessments of pain, as there was no formal procedure to blind clinicians to the LANSS score.

#### 3.2.21 McGill Pain Questionnaire

The McGill Pain Questionnaire was included in a 2019 review (Abahussin et al. 2019), in one article published in 2002. A total of 114 adult cancer inpatients and outpatients were assessed using this questionnaire. Indeterminate evidence was reported for construct and structural validity, as well as for internal consistency and test–retest reliability in a study of poor methodological quality.

#### 3.2.22 Modified Behavioural Pain Scale (MBPS)

The MBPS was evaluated in a systematic review published in 2018 (Crellin et al. 2018), including articles from 1995 to 2015. Twelve studies assessed the scale in 854 infants and children (2 months to 18 years) in inpatient and outpatient settings. Known‐group validity and criterion validity were demonstrated as low evidence in one and four studies, respectively. Very low evidence was also reported for convergent validity by four studies. For internal consistency, low evidence was reported by two studies. The scale's inter‐rater reliability was assessed in seven studies, with contrasting findings. Five studies provided low evidence, while two studies—one of doubtful quality and one of adequate quality—showed moderate‐to‐high evidence. Concerning responsiveness, although seven studies rated it as sufficient, the level of evidence of this property was from low to very low.

#### 3.2.23 Multidimensional Assessment of Pain Scale (MAPS)

The MAPS was evaluated in a systematic review published in 2014 (Dorfman et al. 2014). Two articles, both published in 2007, assessed the scale within a cohort of 63 mechanically ventilated patients in a PICU. Regarding validity, convergent validity was demonstrated as sufficient in two studies of very good and adequate quality. Similarly, criterion validity was confirmed as sufficient, with the same studies. The internal reliability of the MAPS was examined in the same two studies, which reported sufficient evidence for internal consistency but demonstrated a low level of internal reliability due to a lack of reported structural validity. Inter‐rater reliability was demonstrated as sufficient in one study of very good quality. Additionally, responsiveness was assessed and found sufficient evidence in a single study of adequate quality.

#### 3.2.24 Neuropathic Pain Diagnostic Questionnaire (DN4)

The DN4 was evaluated in a 2020 systematic review (Edwards et al. 2020), which included one 2011 study of 19 adult stroke survivors at various recovery stages in a rehabilitation setting. Convergent validity was evaluated in one study, which reported indeterminate evidence and was classified as having inadequate quality.

#### 3.2.25 Nociception Coma Scale (NCS)

The NCS was evaluated in a systematic review published in 2017 (Vink et al. 2017), including articles from 2009 to 2015. A total of five articles assessed the scale in 166 adult patients with disorders of consciousness caused by acquired brain injury across both semi‐acute and long‐term care settings. In terms of validity, content validity showed indeterminate evidence in one study of very good quality. Similarly, cross‐cultural validity was assessed in two studies of inadequate quality, both of which reported indeterminate results. On the other hand, both construct and convergent validity were shown to be sufficient in one study of adequate and very good quality, respectively. The internal consistency of the NCS was insufficient, as evidenced by one study of adequate quality. Similarly, inter‐rater reliability was found to be insufficient in three studies of adequate and doubtful quality. Responsiveness showed sufficient evidence reported in three studies of very good and adequate quality.

#### 3.2.26 Nociception Coma Scale‐Revised (NCR‐r)

The NCR‐r was evaluated in a systematic review published in 2017 (Vink et al. 2017), including five studies conducted between 2012 and 2015 involving 182 adult patients with disorders of consciousness due to acquired brain injury. These studies covered both acute and long‐term care settings, though none included intubated patients. Construct and convergent validity were reported as sufficient and supported in two studies characterised by adequate and doubtful quality, respectively. Internal and inter‐rater reliability, as demonstrated in a study of adequate quality, was both found to be insufficient. Responsiveness of the NCR‐r was rated as sufficient in two studies of very good and doubtful methodological quality.

#### 3.2.27 Numeric Pain Rating Scale (NPRS)

The NPRS was evaluated in five reviews (2019–2022) (Baamer et al. 2022; Chiarotto et al. 2019; Edwards et al. 2020; Modarresi et al. 2021; Caravau et al. 2020) covering articles from 1991 to 2020, assessing 48 studies on over 20,000 adults with diverse conditions, such as cerebral palsy, neck pain, stroke, low back pain, or hospitalisation. Construct validity showed inconsistent and insufficient results in two studies showing very low evidence. Concurrent validity was sufficient in 11 studies of adequate or very good quality, and in one study of inadequate quality, showing mostly a moderate level of evidence, but insufficient in four studies, three of which had adequate quality. Content validity was of low evidence as demonstrated in two studies of adequate quality. Convergent validity was sufficient in six studies, mostly of adequate quality, showing a high level of evidence. Criterion validity showed sufficient results in one study of inadequate quality, while two studies reported inconsistent findings revealing low evidence, and structural validity was rated sufficient but with very low evidence.

The NPRS demonstrated reliability in studies of varying quality, though with risks of bias. One adequate‐quality study provided low evidence for internal reliability. Four inadequate‐quality studies reported sufficient inter‐rater reliability, but with low‐quality evidence due to serious bias risks. Moderate to high test–retest reliability was found in four studies of adequate‐to‐very‐good quality on neck pain patients. However, five studies on low back pain showed inconsistent results, indicating low evidence for test–retest reliability in those patients.

Five studies showed insufficient results for measurement error of the NPRS. Regarding responsiveness, seven studies of adequate to very good quality conducted on patients with neck pain reported sufficient responsiveness of the NPRS. In contrast, nine studies, mostly of doubtful or inadequate quality, reported inconsistent or indeterminate evidence regarding the NPRS's sensitivity to change.

#### 3.2.28 Pain Assessment Scale for Seniors With Severe Dementia‐II (PACSLAC‐II)

The PACSLAC‐II was reviewed in 2020 (Edwards et al. 2020) based on a single study conducted in 2018, which included 36 adult stroke survivors in a stroke unit. The study highlighted sufficient internal reliability.

#### 3.2.29 Pain Evaluation Scale (PES)

The PES was assessed in a systematic review published in 2020 (Caravau et al. 2020), which included two studies conducted between 2001 and 2010, focusing on 408 patients with severe cerebral palsy. The content and construct validity of the PES demonstrated low levels of evidence for these properties, as indicated by findings from one study and two studies, respectively. Internal reliability was rated as indeterminate in two studies of very good quality, and inter‐rater reliability had an insufficient rating in one study of doubtful methodological quality.

#### 3.2.30 PainDETECT (PDQ) Screening Tool

The PDQ screening tool was evaluated in a 2017 review that included two studies published in 2013 and 2015, respectively, with 1409 cancer patients experiencing neuropathic or non‐neuropathic pain (Mulvey et al. 2017). The two studies of adequate quality found that specificity ranged from 77% to 97%, and sensitivity from 18% to 53%.

#### 3.2.31 Paediatric Pain Profile (PPP)

The PPP was reviewed in two systematic reviews published in 2020 (Caravau et al. 2020) and 2023 (Smith et al. 2023), respectively, which included three studies conducted between 2004 and 2013. These studies focused on children with severe cerebral palsy and other neurological conditions or with children aged 1–18 years who were unable to self‐report, with a total sample size of 269. The content and the cross‐cultural validity of the PPP were evaluated in one study providing low and very low evidence for them due to studies of doubtful or inadequate methodological quality. In contrast, construct validity was rated as sufficient in two studies of adequate quality, providing high evidence for this property. Low evidence was demonstrated for internal reliability in three studies due to inconsistency, and no reported structural validity. Contrasting results were found for test–retest reliability: two studies conducted on children unable to self‐report showed high evidence of this property, whereas one study of doubtful methodological quality involving children with severe cerebral palsy demonstrated very low evidence. Finally, the responsiveness of the PPP was rated as sufficient, demonstrating moderate evidence of it.

#### 3.2.32 Questionnaire for Intense Episodic Pain (QUEDI)

The QUEDI was evaluated in a systematic review published in 2021 (Liossi et al. 2021), based on one study conducted in 2012 involving 229 cancer patients in palliative care settings. Test–retest reliability was supported; though the quality of evidence was low.

#### 3.2.33 Questionnaire on Pain Caused by Spasticity (QPS)

The QPS was evaluated in a systematic review published in 2020 (Caravau et al. 2020), including two studies published in 2014 and 2017, respectively, involving 170 children and adolescents with cerebral palsy who experienced limb spasticity‐related pain. Content validity showed moderate evidence and was deemed sufficient in a study of doubtful quality. Construct validity was rated as insufficient in a study of doubtful quality, demonstrating very low evidence for it, while the structural validity of the Chinese version of the QPS resulted as indeterminate, providing moderate evidence of it. The reliability of the QPS was rated as sufficient for both internal and inter‐rater reliability.

#### 3.2.34 Verbal Rating Scale (VRS)

The VRS was evaluated by a total of eight studies included in two systematic reviews published in 2016 (de Vries et al. 2016) and 2022 (Baamer et al. 2022), encompassing research conducted between 1989 and 2011. The patient populations included stroke patients with aphasia (127 patients) and hospitalised adult postoperative inpatients (5167 patients). One study of very good methodological quality presented a sufficient rating result in strong positive evidence for content validity. Five studies of adequate quality and one study of inadequate quality demonstrated high evidence for an inconsistent rating of convergent validity. Criterion validity showed an inconsistent rating in two studies of adequate and inadequate quality, respectively, providing low evidence for this validity. Inter‐rater and intra‐rater reliability were shown as insufficient in one study of doubtful quality. Two studies of inadequate quality demonstrated sufficient test–retest reliability with low evidence. Responsiveness was evaluated in two studies of doubtful and inadequate quality, which reported indeterminate or insufficient responsiveness of the scale, providing low evidence for this property.

#### 3.2.35 Visual Analogue Scale (VAS)

The VAS was evaluated in six reviews (from 2016 to 2022) (Baamer et al. 2022; Chiarotto et al. 2019; Dabbagh et al. 2022; de Vries et al. 2016; Edwards et al. 2020; Modarresi et al. 2021) covering studies from 1982 to 2020, including 46 articles with over 10 000 adults across various conditions, such as ulnar nerve entrapment, neck pain, stroke with aphasia, stroke survivors, low back pain and hospitalised patients.

Regarding content validity, two studies reported mixed results: one rated it as sufficient, while the other rated it as inconsistent, suggesting low evidence for content validity. Regarding construct validity, one very good‐quality study reported a sufficient rating, while two studies showed an inconsistent rating, leading to low‐quality evidence for this property. Seven studies presented sufficient ratings for concurrent validity, showing moderate evidence for this validity. For convergent validity, eight studies supported its validity, rated as sufficient with a high level of evidence. In terms of criterion validity, two studies showed sufficient ratings, while another of inadequate methodological quality reported an indeterminate rating. One study showed very low‐quality evidence for cross‐cultural validity, providing an indeterminate rating for a Ghanaian version of the VAS.

Inter‐rater reliability was inconclusive: two studies of doubtful quality rated it as sufficient, while two studies of inadequate quality reported it as insufficient. Test–retest reliability was assessed in four studies, showing sufficient results, but the evidence was rated as low and very low due to significant risk of bias. Regarding measurement error, three studies rated it as indeterminate and inconsistent. Responsiveness was assessed in 12 studies. Only in two studies was the VAS's responsiveness rated as sufficient, one of these studies being of very good quality. In the remaining studies, responsiveness was rated as inconsistent or indeterminate, providing low evidence for this psychometric property.

#### 3.2.36 Webber's Breakthrough Cancer Pain Algorithm

The Webber's Breakthrough Cancer Pain Algorithm was reviewed in 2021 (Liossi et al. 2021), with one study conducted in 2013 involving 135 cancer patients in hospital and hospice settings. Content validity and known‐group validity were rated as inconsistent or indeterminate, providing for both properties evidence of very low or low quality. Criterion validity was reported as insufficient. Sensitivity varied significantly across subgroups, ranging from 0.36 to 0.66 depending on patient characteristics.

#### 3.2.37 6‐Point Faces Pain Scale (FPS‐6)

The FPS‐6 was analysed in four reviews published from 2016 to 2022 in 10 articles published from 2003 to 2015 (Baamer et al. 2022; Caravau et al. 2020; de Vries et al. 2016; Edwards et al. 2020). A total of 1312 patients—including those with cerebral palsy, stroke (with or without aphasia) in rehabilitation settings or hospitalised surgical patients—were assessed using the FPS‐6. As for the validity of the FPS‐6, content, criterion, convergent, construct and structural validity were supported as sufficient in all the studies, although with different levels of methodological quality. Regarding inter‐rater reliability, one study provided insufficient evidence, two studies provided indeterminate evidence, and four studies provided sufficient evidence but were of inadequate methodological quality, so the level of evidence was low. The same four studies also reported sufficient evidence for test–retest reliability but were similarly of a low level of evidence. Responsiveness was demonstrated as sufficient in one study of doubtful quality, while four studies of inadequate or doubtful methodological quality provided indeterminate evidence.

#### 3.2.38 7‐Point Faces Pain Scale (FPS‐7), 16‐Point Verbal Rating Scale (VRS‐16), 21‐Point Numeric Rating Scale (NRS‐21), 5‐Point Verbal Rating Scale (VRS‐5)

All these scales were examined in a 2020 review (Caravau et al. 2020), which included one study published in 2003 involving 69 individuals with cerebral palsy. The same study and sample were used for all the scales. The FPS‐7, VRS‐16 and NRS‐21 demonstrated sufficient structural and construct validity. However, the methodological quality of the study was rated as adequate for structural validity but inadequate for construct validity. The evidence for structural validity was moderate for the FPS‐7 and low for the VRS‐16 and NRS‐21, while construct validity evidence was rated as very low for all three scales. In contrast, the VRS‐5's structural validity was rated as sufficient, but its construct validity was insufficient.

### 3.3 Overlap Between Included Systematic Reviews

The overlap percentage was 5% and the CCA 0.003. These results indicate a slight overlap among the included reviews, suggesting that the systematic reviews addressed different studies related to psychometric properties of the scales.

### 3.4 Assessment of Methodological Quality of the Studies

The synthesis of this assessment is displayed in Table 3, offering a detailed overview of the quality ratings, strengths, and limitations of the systematic reviews based on the JBI checklist.

**TABLE 3:** JBI critical appraisal checklist for systematic reviews and research syntheses.

	Dabbagh et al. (2022)	Dorfman et al. (2014)	Abahussin et al. (2019)	Caravau et al. (2020)	de Vries et al. (2016)	Liossi et al. (2021)	Modarresi et al. (2021)	Vink et al. (2017)	Maaskant et al. (2016)	Edwards et al. (2020)	Baamer et al. (2022)	Crellin et al. (2015)	Mulvey et al. (2017)	Crellin et al. (2018)	Zhai et al. (2020)	Chiarotto et al. (2019)	Smith et al. (2023)
1. Is the review question clearly and explicitly stated?	Yes	Yes	Yes	Yes	Yes	Yes	Yes	Yes	Yes	Yes	Yes	Yes	Yes	Yes	Yes	Yes	Yes
2. Were the inclusion criteria appropriate for the review question?	Yes	Yes	Yes	Yes	Yes	Yes	Yes	Yes	Yes	Yes	Yes	Yes	Yes	Yes	Yes	Yes	Yes
3. Was the search strategy appropriate?	Yes	Yes	Yes	Yes	Yes	Yes	Yes	Yes	Yes	Yes	Yes	Yes	Yes	Yes	Yes	Yes	Yes
4. Were the sources and resources used to search for studies adequate?	Yes	Yes	Yes	Yes	Yes	Yes	Yes	Yes	Yes	Yes	Yes	Yes	Yes	Yes	Yes	Yes	Yes
5. Were the criteria for appraising studies appropriate?	Yes	Yes	Yes	Yes	Yes	Yes	Yes	Yes	Yes	Yes	Yes	Yes	Yes	Yes	Yes	Yes	Yes
6. Was critical appraisal conducted by two or more reviewers independently?	Yes	Yes	Yes	Yes	Yes	Yes	Yes	Yes	Yes	Yes	Yes	Yes	Yes	Yes	Yes	Yes	Yes
7. Were there methods to minimise errors in data extraction?	Yes	Yes	Yes	Yes	Yes	Yes	Yes	Yes	Yes	Yes	Yes	Yes	Yes	Yes	Yes	Yes	Yes
8. Were the methods used to combine studies appropriate?	Yes	Yes	Yes	Yes	Yes	Yes	Yes	Yes	Yes	Yes	Yes	Yes	Yes	Yes	Yes	Yes	Yes
9. Was the likelihood of publication bias assessed?	No	No	No	No	No	No	No	No	No	No	No	No	No	No	No	No	No
10. Were recommendations for policy and/or practice supported by the reported data?	Yes	Yes	Yes	Yes	Yes	Yes	Yes	Yes	Yes	Yes	Yes	Yes	Yes	Yes	Yes	Yes	Yes
11. Were the specific directives for new research appropriate?	Yes	Yes	Yes	Yes	Yes	Yes	Yes	Yes	Yes	Yes	Yes	Yes	Yes	Yes	Yes	Yes	Yes
Overall Score	10/11 (91%)	10/11 (91%)	10/11 (91%)	10/11 (91%)	10/11 (91%)	10/11 (91%)	10/11 (91%)	10/11 (91%)	10/11 (91%)	10/11 (91%)	10/11 (91%)	10/11 (91%)	10/11 (91%)	10/11 (91%)	10/11 (91%)	10/11 (91%)	10/11 (91%)
Overall Quality	HIGH	HIGH	HIGH	HIGH	HIGH	HIGH	HIGH	HIGH	HIGH	HIGH	HIGH	HIGH	HIGH	HIGH	HIGH	HIGH	HIGH

## 4 Discussion

This umbrella review identified pain scales that are reliable for nurses' use in hospitalised patients, determining which ones have the best psychometric properties and can be recommended for clinical use. Overall, the quality of the reviews included was high, ensuring reliable findings. To discuss the results, we grouped the scales into three categories based on the COSMIN guidelines (Prinsen et al. 2018), after considering content validity as the most critical psychometric property.

### 4.1 Scales With Sufficient Content Validity and at Least Low‐Quality Evidence for Sufficient Internal Consistency: Recommended for Use

Three scales, tested in two different patient populations, met the criteria for this category. For adult cancer patients, the BAT showed sufficient content, structural and construct validity, despite the level of evidence being low for content validity and moderate for the other validity properties. BAT's reliability was also sufficient, with a high level of evidence for internal consistency as well as for its responsiveness. Additionally, the BAT was easy to administer and score. Unfortunately, these good results were found in only one article; while to claim that psychometrics have been well established, the measure must have been presented in at least two studies by different research teams (Cohen et al. 2008). Other pain scales tested for this population either lacked content validity assessment or were not rated as sufficient. This may stem from researchers assuming established scales did not require validation or from using scales not designed for cancer patients, compromising pain assessment and management in this vulnerable population (Cluxton 2019).

For paediatric patients, we found two scales that fall into this category. The QPS, used for patients with limb spasticity pain, demonstrated sufficient content validity, internal consistency, and inter‐rater reliability, while evidence for construct and structural validity was not found. Two studies tested the psychometric properties of the QPS, but each property was tested in only one study, requiring further research to support these findings. Overall, three studies tested the psychometric properties of the PPP, used for paediatric patients who are unable to self‐report, providing evidence to support its use in clinical practice. The PPP demonstrated a low level of evidence for sufficient content validity and internal consistency, also showing sufficient construct validity and responsiveness. Test–retest reliability was supported in two studies involving children unable to self‐report, while another study of doubtful quality, conducted on children with severe cerebral palsy, rated it as indeterminate. In the paediatric population, the FLACC scale was assessed for content validity, but with insufficient results. These findings show that pain can be particularly challenging to assess in children, resulting in unrelieved or under‐treated symptomatology (Manocha and Taneja 2016), highlighting the need for further studies in this population.

### 4.2 Scales With High‐Quality Evidence Indicating an Insufficient Measurement Property: Not Recommended for Use

Two scales met the criteria for this category. The Webber's Breakthrough Cancer Pain Algorithm, showed a high level of evidence of insufficient criterion validity. Overall, its psychometric properties were not adequate along with its poor diagnostic sensitivity. Finally, despite the algorithm's ease of administration, it proved difficult for both patients and clinicians to understand. These results indicate that its utility in helping to pinpoint and control breakthrough pain is questionable.

The NPRS showed a high level of evidence for an insufficient rating in measurement error across five studies. Despite being one of the most widely used and studied scales in different populations, its psychometric properties yielded mixed results. This is likely due to its unidimensional nature, which may require complementary measurements to accurately assess patient pain. However, its simplicity and likelihood of consistent use make it a practical tool for enhancing decision‐making and care quality (Kroenke 2018).

### 4.3 Scales That Do Not Fit Into Either Category (1) or (2): Potential to Be Recommended for Use

Most of the scales (*n* = 36) fall into this category, suggesting further evaluation before their recommendation for clinical practice. Within this category, we distinguished three subgroups, as follows.

#### 4.3.1 Scales With Sufficient Evidence of Content Validity

The Doloplus‐2 scale, FPS‐6, VAS and VRS demonstrated sufficient content validity. Among these, the FPS‐6 and VAS also showed sufficient forms of validity. However, internal consistency was not assessed due to their unidimensional nature. The FPS‐6's inter‐rater reliability, test–retest reliability, and responsiveness had low evidence, while the VAS's test–retest reliability showed low evidence, and its inter‐rater reliability yielded inconclusive results in stroke survivors. Additionally, the VAS had limitations in responsiveness and measurement error, with inconclusive findings. Despite these limitations, both the FPS‐6 and VAS appear to be adequate tools, though further studies are needed. The VAS is one of the two most studied scales in the literature, with 46 articles and over 10,000 adult patients. It is easy to implement in clinical practice, and its scores are straightforward to interpret. However, it may be more effective when combined with multidimensional tools to capture the complexity of pain. In contrast, the Doloplus‐2 scale and the VRS generally lacked adequate psychometric properties and required further refinement.

#### 4.3.2 Scales With Sufficient Evidence of Psychometric Properties but no Reported Content Validity

Fourteen scales demonstrated sufficient psychometric properties, most of which were designed for the paediatric population. These include the mFLACC, MAPS, COMFORT‐n, COMFORT‐w, mBPI, COMFORT and COMFORT‐b. Most of these scales were tested in only one study, except for the MAPS, which was analysed in two studies; and the COMFORT and COMFORT‐b, which were analysed in a total of nine studies each. Among these, the two COMFORT scales stand out as having the most robust psychometric properties. They have been extensively studied in multiple large‐sample studies involving neonates and paediatric patients. Additionally, they are easy to implement in nursing practice.

The FPS‐7, VRS‐16, and NRS‐21 were tested in a single study of individuals with verbal communication disorders and cerebral palsy. Although their validity was sufficient, the level of evidence was low; reliability was not assessed. Furthermore, reliance on a single study reduces confidence in the findings.

Two scales, the BPI‐SF and QUEDI, were analysed in inpatient, outpatient, and palliative cancer patients. However, for the QUEDI, only test–retest reliability was reported in a single study; no other psychometric properties were assessed. In contrast, the BPI‐SF demonstrated sufficient psychometric properties across three studies involving adult cancer inpatients and outpatients. Although most of these studies were of doubtful quality, the BPI‐SF appears to be a promising scale for use in this population. Finally, the PACSLAC‐II was tested in only one study with a small sample of stroke patients. While reliability was demonstrated, validity was not evaluated, and the limited sample size reduced the generalisability of the findings.

Overall, although the COMFORT, COMFORT‐b, and BPI‐SF demonstrated the most robust psychometric properties, studies on their content validity are needed to support their use in clinical practice.

#### 4.3.3 Scales With Inconsistent, Indeterminate, or Insufficient Evidence

Nineteen scales fell into this category, representing nearly half of those identified in this review, due to inconsistent, indeterminate, or insufficient evidence for their psychometric properties, likely caused by methodological issues, small sample sizes, or statistical methods. Without further validation to resolve these discrepancies, their use in clinical practice remains questionable. These findings highlight the need for high‐quality studies to establish the psychometric properties of these instruments before they can be recommended for clinical use.

Finally, it should be noted that there are scales for which this review did not find sufficiently robust evidence to recommend their use (i.e., categories 2 and 3); yet they remain part of routine clinical practice, such as the VAS. Further psychometric testing is warranted to support their continued use or to facilitate their replacement with more robust measures (Prinsen et al. 2018).

## 5 Limitations

This review has some limitations. The included studies were heterogeneous for a unified meta‐analysis. While most studies used the COSMIN checklist, some did not report evidence quality using the GRADE framework, or used different appraisal tools, potentially contributing to heterogeneity in results. Additionally, the search strategy included terms related to hospitalisation, which may have excluded relevant reviews and scales, introducing potential selection bias.

## 6 Conclusion

The PPP is recommended for paediatric patients unable to self‐report; the BAT for cancer patients, and the QPS for paediatric patients with limb spasticity are also recommended, although the positive results for both the BAT and QPS should be confirmed by at least one additional study. Most scales have the potential to be recommended for use, although further studies are needed to support such recommendations. In hospital clinical practice, nurses are expected to use multidimensional scales to better capture the complexity of pain. Therefore, in settings where unidimensional tools such as the VAS are used, these should complement rather than replace multidimensional instruments in order to ensure a comprehensive assessment.

Further studies are needed to refine or develop scales with robust psychometric properties. This review supports standardising documentation with validated scales to enhance care quality, enable secondary data use, and improve pain assessment and patient outcomes.

## Author Contributions

Antonello Cocchieri and Fabio D'Agostino: Conceptualisation, data curation, formal analysis, methodology, supervision, project administration, funding acquisition, writing – original draft, writing – review and editing; Claudia Fantuzzi: methodology, supervision, writing – original draft, writing – review and editing; Daniela D'Angelo: methodology, supervision, writing – review and editing; Gianfranco Sanson: Conceptualisation, methodology, supervision, writing – original draft, writing – review and editing; Manuele Cesare: methodology, supervision, visualisation, writing – original draft, writing – review and editing; Noemi Giannetta: Methodology, supervision, visualisation, writing – original draft, writing – review and editing; Sara Cignola: Methodology, supervision, writing – original draft, writing – review and editing.

## Ethics Statement

The authors have nothing to report.

## Conflicts of Interest

The authors declare no conflicts of interest.

## Supporting information


**Appendix S1:** jocn70071‐sup‐0001‐AppendixS1.pdf.

**Appendix S2:** Summary of psychometric characteristic of scales included.

## Data Availability

The authors have nothing to report.
