# Supplementary material for: Psychometric Properties of Pain Scales in Inpatient Settings: An Umbrella Review
Source: J Clin Nurs. 2025 Aug 18;35(3):1002–36. doi: 10.1111/jocn.70071 (PMC12862562; doi:10.1111/jocn.70071)
Supplement: Supplementary file 2 — Appendix S2: Summary of psychometric characteristic of scales included. [file JOCN-35-1002-s002.docx]

|  |
| --- |

| **Appendix S2.** Summary of psychometric characteristic of scales included. | | | | | | | | | | | | | | | | | | | | | | | | | | | | | | | | | | | | | | | | | | |
| --- | --- | --- | --- | --- | --- | --- | --- | --- | --- | --- | --- | --- | --- | --- | --- | --- | --- | --- | --- | --- | --- | --- | --- | --- | --- | --- | --- | --- | --- | --- | --- | --- | --- | --- | --- | --- | --- | --- | --- | --- | --- | --- |
| **Outcome measure** | | | **BAT** | **BAT-DL** | **BAT-K** | **BPI** | | | | | **mBPI** | **BPI-PS** | | **BPI-SF** | | | | **CPI** | **CAAS** | **COMFORT** | | | **COMFORT – b** | | | **COMFORT-n** | **COMFORT-w** | **CPOT** | **Doloplus-2** | **FLACC** | | **mFLACC** | **revised-FLACC** | | **IQ-BTP** | **LANSS** | **McGill Pain Quest.** | **MBPS** | | | | |
| COSMIN Grade* | | | A^1^ | B | B | B | | | | | B | B | | B | | | | B | B | B | | | B | | | B | B | B | B | B | | B | B | | B | B | B | B | | | | |
|  | |  |  |  |  |  |  |  |  |  |  |  |  |  |  |  |  |  |  |  |  |  |  |  |  |  |  |  |  |  |  |  |  |  |  |  |  |  |  |  |  |  |
| Content | Nr of studies | | 1 | 1 | 1 |  |  |  |  | |  | 1 |  |  | |  |  |  |  |  |  |  |  |  |  |  |  |  | 1 | 2 |  |  |  |  | 1 |  |  |  |  |  |  |  |
| validity | ROB score | | I | D | D |  |  |  |  | |  | A |  |  | |  |  |  |  |  |  |  |  |  |  |  |  |  | I | I |  |  |  |  | I |  |  |  |  |  |  |  |
|  | Rating | | + | ? | ? |  |  |  |  | |  | - |  |  | |  |  |  |  |  |  |  |  |  |  |  |  |  | + | - |  |  |  |  | - |  |  |  |  |  |  |  |
|  | Quality | | L | L | L |  |  |  |  | |  | L |  |  | |  |  |  |  |  |  |  |  |  |  |  |  |  | VL | VL |  |  |  |  | L |  |  |  |  |  |  |  |
|  |  | |  |  |  |  |  |  |  | |  |  |  |  | |  |  |  |  |  |  |  |  |  |  |  |  |  |  |  |  |  |  |  |  |  |  |  |  |  |  |  |
| Structural | Nr of studies | | 1 | 1 | 1 | 3 | 1 | 3 | 1 | |  | 1 |  | 1 | | 2 |  | 1 |  |  |  |  |  |  |  |  |  |  | 1 | 4 |  |  |  |  | 1 |  | 1 |  |  |  |  |  |
| validity | ROB score | | A | V | A | D | I | D | V | |  | A |  | A | | D |  | D |  |  |  |  |  |  |  |  |  |  | I | / |  |  |  |  | A |  | I |  |  |  |  |  |
|  | Rating | | + | ? | ? | - | - | ? | ? | |  | + |  | ? | | + |  | ? |  |  |  |  |  |  |  |  |  |  | ? | / |  |  |  |  | ? |  | ? |  |  |  |  |  |
|  | Quality | | M | H | M |  |  |  |  | |  | M |  |  | |  |  |  |  |  |  |  |  |  |  |  |  |  | VL | M |  |  |  |  | M |  | VL |  |  |  |  |  |
|  |  | |  |  |  |  |  |  |  | |  |  |  |  | |  |  |  |  |  |  |  |  |  |  |  |  |  |  |  |  |  |  |  |  |  |  |  |  |  |  |  |
| Construct | Nr of studies | | 1 | 1 |  | 4 | 2 |  |  | | 1 | 1 | 1 | 1 | | 2 |  | 1 |  | 1 | 1 | 2 | 5 | 1 |  | 1 | 1 |  | 1 | 11 | 4 | 1 | 1 |  | 1 |  | 1 |  |  |  |  |  |
| validity | ROB score | | V | V |  | I | D |  |  | | V | V | A | I | | D |  | D |  | A | I | D | D | A |  | D | I |  | A | / | / | D | D |  | V |  | I |  |  |  |  |  |
|  | Rating | | + | + |  | + | + |  |  | | + |  | - | + | | + |  | + |  | + | - | + | + | + |  | + | + |  | + | / | / | + | + |  | + |  | ? |  |  |  |  |  |
|  | Quality | | M | M |  |  |  |  |  | | H |  | M |  | |  |  |  |  |  |  |  |  |  |  |  |  |  | M | M | VL |  | L |  | H |  | VL |  |  |  |  |  |
|  |  | |  |  |  |  |  |  |  | |  |  |  |  | |  |  |  |  |  |  |  |  |  |  |  |  |  |  |  |  |  |  |  |  |  |  |  |  |  |  |  |
| Convergent | Nr of studies | |  |  |  |  |  |  |  | |  |  |  |  | |  |  |  |  |  |  |  |  |  |  |  |  |  |  |  |  |  |  |  |  |  |  | 2 | 1 | 1 |  |  |
| validity | ROB score | |  |  |  |  |  |  |  | |  |  |  |  | |  |  |  |  |  |  |  |  |  |  |  |  |  |  |  |  |  |  |  |  |  |  | I | I | D |  |  |
|  | Rating | |  |  |  |  |  |  |  | |  |  |  |  | |  |  |  |  |  |  |  |  |  |  |  |  |  |  |  |  |  |  |  |  |  |  | - | + | - |  |  |
|  | Quality | |  |  |  |  |  |  |  | |  |  |  |  | |  |  |  |  |  |  |  |  |  |  |  |  |  |  |  |  |  |  |  |  |  |  | VL | VL | VL |  |  |
|  |  | |  |  |  |  |  |  |  | |  |  |  |  | |  |  |  |  |  |  |  |  |  |  |  |  |  |  |  |  |  |  |  |  |  |  |  |  |  |  |  |
| Criterion | Nr of studies | |  |  |  |  |  |  |  | |  |  |  |  | |  |  |  | 1 |  |  |  | 1 |  |  |  |  |  |  | 5 | 6 | 1 | 1 |  |  |  |  | 4 |  |  |  |  |
| validity | ROB score | |  |  |  |  |  |  |  | |  |  |  |  | |  |  |  | D |  |  |  | V |  |  |  |  |  |  | / | / | A | V |  |  |  |  | I |  |  |  |  |
|  | Rating | |  |  |  |  |  |  |  | |  |  |  |  | |  |  |  | - |  |  |  | + |  |  |  |  |  |  | / | / | + | ? |  |  |  |  | + |  |  |  |  |
|  | Quality | |  |  |  |  |  |  |  | |  |  |  |  | |  |  |  |  |  |  |  |  |  |  |  |  |  |  | M | VL |  | L |  |  |  |  | L |  |  |  |  |
|  |  | |  |  |  |  |  |  |  | |  |  |  |  | |  |  |  |  |  |  |  |  |  |  |  |  |  |  |  |  |  |  |  |  |  |  |  |  |  |  |  |
| Cross cultural validity/ | Nr of studies | |  |  |  |  |  |  |  | |  |  |  |  | |  |  |  |  |  |  |  |  |  |  |  |  |  | 1 |  |  |  |  |  |  |  |  |  |  |  |  |  |
| measurement invariance | ROB score | |  |  |  |  |  |  |  | |  |  |  |  | |  |  |  |  |  |  |  |  |  |  |  |  |  | I |  |  |  |  |  |  |  |  |  |  |  |  |  |
|  | Rating | |  |  |  |  |  |  |  | |  |  |  |  | |  |  |  |  |  |  |  |  |  |  |  |  |  | ? |  |  |  |  |  |  |  |  |  |  |  |  |  |
|  | Quality | |  |  |  |  |  |  |  | |  |  |  |  | |  |  |  |  |  |  |  |  |  |  |  |  |  | VL |  |  |  |  |  |  |  |  |  |  |  |  |  |
|  |  | |  |  |  |  |  |  |  | |  |  |  |  | |  |  |  |  |  |  |  |  |  |  |  |  |  |  |  |  |  |  |  |  |  |  |  |  |  |  |  |
| Known-Group | Nr of studies | |  |  |  |  |  |  |  | |  |  |  |  | |  |  |  |  |  |  |  |  |  |  |  |  |  |  |  |  |  |  |  |  |  |  | 1 |  |  |  |  |
| Validity | ROB score | |  |  |  |  |  |  |  | |  |  |  |  | |  |  |  |  |  |  |  |  |  |  |  |  |  |  |  |  |  |  |  |  |  |  | D |  |  |  |  |
|  | Rating | |  |  |  |  |  |  |  | |  |  |  |  | |  |  |  |  |  |  |  |  |  |  |  |  |  |  |  |  |  |  |  |  |  |  | + |  |  |  |  |
|  | Quality | |  |  |  |  |  |  |  | |  |  |  |  | |  |  |  |  |  |  |  |  |  |  |  |  |  |  |  |  |  |  |  |  |  |  | L |  |  |  |  |
|  |  | |  |  |  |  |  |  |  | |  |  |  |  | |  |  |  |  |  |  |  |  |  |  |  |  |  |  |  |  |  |  |  |  |  |  |  |  |  |  |  |
| Concurrent | Nr of studies | |  |  |  |  |  |  |  | |  |  |  |  | |  |  |  |  |  |  |  |  |  |  |  |  |  |  |  |  |  |  |  |  |  |  |  |  |  |  |  |
| Validity | ROB score | |  |  |  |  |  |  |  | |  |  |  |  | |  |  |  |  |  |  |  |  |  |  |  |  |  |  |  |  |  |  |  |  |  |  |  |  |  |  |  |
|  | Rating | |  |  |  |  |  |  |  | |  |  |  |  | |  |  |  |  |  |  |  |  |  |  |  |  |  |  |  |  |  |  |  |  |  |  |  |  |  |  |  |
|  | Quality | |  |  |  |  |  |  |  | |  |  |  |  | |  |  |  |  |  |  |  |  |  |  |  |  |  |  |  |  |  |  |  |  |  |  |  |  |  |  |  |
|  |  | |  |  |  |  |  |  |  | |  |  |  |  | |  |  |  |  |  |  |  |  |  |  |  |  |  |  |  |  |  |  |  |  |  |  |  |  |  |  |  |
| Internal | Nr of studies | | 1 | 1 | 1 | 4 | 1 | 2 | 1 | | 1 | 2 |  | 1 | | 1 | 1 | 1 |  | 3 | 1 | 1 | 3 | 1 |  | 1 | 1 |  | 1 | 4 | 2 |  | 1 |  | 1 |  | 1 | 1 | 1 |  |  |  |
| consistency | ROB score | | V | V | V | D | I | A | V | | D | A |  | A | | D | D | D |  | A | D | I | A | D |  | D | A |  | V | / | / |  | V |  | A |  | I | D | I |  |  |  |
|  | Rating | | + | ? | + | + | + | + | + | | + | + |  | + | | + | ? | - |  | + | + | + | + | + |  | + | + |  | ? | / | / |  | + |  | + |  | ? | - | + |  |  |  |
|  | Quality | | H | H | H |  |  |  |  | | L | M |  |  | |  |  |  |  |  |  |  |  |  |  |  |  |  | VL | M | VL |  | M |  | H |  | VL | L | L |  |  |  |
|  |  | |  |  |  |  |  |  |  | |  |  |  |  | |  |  |  |  |  |  |  |  |  |  |  |  |  |  |  |  |  |  |  |  |  |  |  |  |  |  |  |
| Inter-Rater Reliability | Nr of studies | |  |  |  | 1 |  |  |  | |  |  |  |  | |  |  |  | 1 | 1 | 1 | 1 | 1 | 3 | 1 | 1 | 1 |  | 2 | 14 | 3 | 1 | 1 | 1 |  |  |  | 1 | 1 | 1 | 2 | 2 |
|  | ROB score | |  |  |  | V |  |  |  | |  |  |  |  | |  |  |  | D | A | V | D | V | A | D | A | D |  | A | / | / | A | V | D |  |  |  | A | D | D | I | I |
|  | Rating | |  |  |  | I |  |  |  | |  |  |  |  | |  |  |  | + | + | + | + | + | + | + | + | + |  | + | / | / | + | - | + |  |  |  | + | + | - | + | - |
|  | Quality | |  |  |  | L |  |  |  | |  |  |  |  | |  |  |  |  |  |  |  |  |  |  |  |  |  | H | M | VL |  |  |  |  |  |  | H | M | L | L | L |
|  |  | |  |  |  |  |  |  |  | |  |  |  |  | |  |  |  |  |  |  |  |  |  |  |  |  |  |  |  |  |  |  |  |  |  |  |  |  |  |  |  |
| Test-retest Reliability |  | | 1 | 1 | 1 | 1 |  |  |  | |  |  |  |  | |  |  |  |  |  |  |  |  |  |  |  |  |  |  |  |  |  |  |  |  |  | 1 |  |  |  |  |  |
|  |  | | D | D | D | I |  |  |  | |  |  |  |  | |  |  |  |  |  |  |  |  |  |  |  |  |  |  |  |  |  |  |  |  |  | I |  |  |  |  |  |
|  |  | | + | + | + | - |  |  |  | |  |  |  |  | |  |  |  |  |  |  |  |  |  |  |  |  |  |  |  |  |  |  |  |  |  | ? |  |  |  |  |  |
|  |  | | L | L | L |  |  |  |  | |  |  |  |  | |  |  |  |  |  |  |  |  |  |  |  |  |  |  |  |  |  |  |  |  |  | VL |  |  |  |  |  |
|  |  | |  |  |  |  |  |  |  | |  |  |  |  | |  |  |  |  |  |  |  |  |  |  |  |  |  |  |  |  |  |  |  |  |  |  |  |  |  |  |  |
| Measurement error | Nr of studies | |  |  |  |  |  |  |  | |  |  |  |  | |  |  |  |  |  |  |  |  |  |  |  |  |  |  |  |  |  |  |  |  |  |  |  |  |  |  |  |
|  | ROB score | |  |  |  |  |  |  |  | |  |  |  |  | |  |  |  |  |  |  |  |  |  |  |  |  |  |  |  |  |  |  |  |  |  |  |  |  |  |  |  |
|  | Rating | |  |  |  |  |  |  |  | |  |  |  |  | |  |  |  |  |  |  |  |  |  |  |  |  |  |  |  |  |  |  |  |  |  |  |  |  |  |  |  |
|  | Quality | |  |  |  |  |  |  |  | |  |  |  |  | |  |  |  |  |  |  |  |  |  |  |  |  |  |  |  |  |  |  |  |  |  |  |  |  |  |  |  |
|  |  | |  |  |  |  |  |  |  | |  |  |  |  | |  |  |  |  |  |  |  |  |  |  |  |  |  |  |  |  |  |  |  |  |  |  |  |  |  |  |  |
| Responsiveness | Nr of studies | | 1 | 1 | 1 |  |  |  |  | |  | 2 |  |  | |  |  |  | 1 | 1 | 2 | 1 | 1 | 1 |  | 1 | 1 |  |  | 12 | 8 |  |  |  |  |  |  | 5 | 2 |  |  |  |
|  | ROB score | | V | I | A |  |  |  |  | |  | I |  |  | |  |  |  | D | V | A | D | A | D |  | V | A |  |  | / | / |  |  |  |  |  |  | D | I |  |  |  |
|  | Rating | | + | + | ? |  |  |  |  | |  |  |  |  | |  |  |  | + | + | + | + | + | + |  | + | + |  |  | / | / |  |  |  |  |  |  | + | + |  |  |  |
|  | Quality | | H | VL | M |  |  |  |  | |  |  |  |  | |  |  |  |  |  |  |  |  |  |  |  |  |  |  | VL | M |  |  |  |  |  |  | L | VL |  |  |  |
|  |  | |  |  |  |  |  |  |  | |  |  |  |  | |  |  |  |  |  |  |  |  |  |  |  |  |  |  |  |  |  |  |  |  |  |  |  |  |  |  |  |
| Sensitivity | Nr of studies | |  |  |  |  |  |  |  | |  |  |  |  | |  |  |  |  |  |  |  |  |  |  |  | 1 | 25 |  |  |  |  |  |  |  | 4 |  |  |  |  |  |  |
|  | ROB score | |  |  |  |  |  |  |  | |  |  |  |  | |  |  |  |  |  |  |  |  |  |  |  |  | D |  |  |  |  |  |  |  | A |  |  |  |  |  |  |
|  | Range (%) | |  |  |  |  |  |  |  | |  |  |  |  | |  |  |  |  |  |  |  |  |  |  |  | 93 | 74,3 |  |  |  |  |  |  |  | 86 - 29.5 |  |  |  |  |  |  |
|  | Quality | |  |  |  |  |  |  |  | |  |  |  |  | |  |  |  |  |  |  |  |  |  |  |  |  |  |  |  |  |  |  |  |  |  |  |  |  |  |  |  |
|  |  | |  |  |  |  |  |  |  | |  |  |  |  | |  |  |  |  |  |  |  |  |  |  |  |  |  |  |  |  |  |  |  |  |  |  |  |  |  |  |  |
| Specificity | Nr of studies | |  |  |  |  |  |  |  | |  |  |  |  | |  |  |  |  |  |  |  |  |  |  |  | 1 | 25 |  |  |  |  |  |  |  | 4 |  |  |  |  |  |  |
|  | ROB score | |  |  |  |  |  |  |  | |  |  |  |  | |  |  |  |  |  |  |  |  |  |  |  |  | D |  |  |  |  |  |  |  | A |  |  |  |  |  |  |
|  | Range (%) | |  |  |  |  |  |  |  | |  |  |  |  | |  |  |  |  |  |  |  |  |  |  |  | 80 | 76,8 |  |  |  |  |  |  |  | 100 - 91 |  |  |  |  |  |  |
|  | Quality | |  |  |  |  |  |  |  | |  |  |  |  | |  |  |  |  |  |  |  |  |  |  |  |  |  |  |  |  |  |  |  |  |  |  |  |  |  |  |  |

| **Outcome measure** | | **MAPS** | | **DN4** | **NCS** | | **NCS - r** | | **NPRS** | | | | | **PACSLAC-II** | **PES** | **PDQ** | **PPP** | | **QUEDI** | **QPS** | **VRS** | | **VAS** | | | | | | | **Webber's** | **FPS-6** | | | **FPS-7** | **VRS-16** | **NRS-21** | **VRS-5** |  |
| --- | --- | --- | --- | --- | --- | --- | --- | --- | --- | --- | --- | --- | --- | --- | --- | --- | --- | --- | --- | --- | --- | --- | --- | --- | --- | --- | --- | --- | --- | --- | --- | --- | --- | --- | --- | --- | --- | --- |
|  |  |  |  |  |  |  |  |  |  |  |  |  |  |  |  |  |  |  |  |  |  |  |  |  |  |  |  |  |  |  |  |  |  |  |  |  |  |  |
| COSMIN Grade* | | B | | B | B | | B | | C | | | | | B | B | B | A | | B | A^1^ | B | | B | | | | | | | C | B | | | B | B | B | B |  |
|  |  |  |  |  |  |  |  |  |  |  |  |  |  |  |  |  |  |  |  |  |  |  |  |  |  |  |  |  |  |  |  |  |  |  |  |  |  |  |
| Content | Nr of studies |  |  |  | 1 |  |  |  | 2 |  |  |  |  |  | 1 |  | 1 |  |  | 1 | 1 |  | 1 | 1 |  |  |  |  |  | 1 | 1 |  |  |  |  |  |  |  |
| validity | ROB score |  |  |  | V |  |  |  | A |  |  |  |  |  | D |  | D |  |  | D | V |  | V | A |  |  |  |  |  | I | V |  |  |  |  |  |  |  |
|  | Rating |  |  |  | ? |  |  |  | - |  |  |  |  |  | ? |  | + |  |  | + | + |  | + | - |  |  |  |  |  | - | + |  |  |  |  |  |  |  |
|  | Quality |  |  |  |  |  |  |  | L |  |  |  |  |  | L |  | L |  |  | M |  |  |  | L |  |  |  |  |  | VL | M |  |  |  |  |  |  |  |
|  |  |  |  |  |  |  |  |  |  |  |  |  |  |  |  |  |  |  |  |  |  |  |  |  |  |  |  |  |  |  |  |  |  |  |  |  |  |  |
| Structural | Nr of studies |  |  |  |  |  |  |  | 1 |  |  |  |  |  |  |  |  |  |  | 1 |  |  |  |  |  |  |  |  |  |  | 1 |  |  | 1 | 1 | 1 | 1 |  |
| validity | ROB score |  |  |  |  |  |  |  | A |  |  |  |  |  |  |  |  |  |  | A |  |  |  |  |  |  |  |  |  |  | A |  |  | A | A | A | A |  |
|  | Rating |  |  |  |  |  |  |  | + |  |  |  |  |  |  |  |  |  |  | ? |  |  |  |  |  |  |  |  |  |  | + |  |  | + | + | + | + |  |
|  | Quality |  |  |  |  |  |  |  | VL |  |  |  |  |  |  |  |  |  |  | M |  |  |  |  |  |  |  |  |  |  | M |  |  | M | L | L | L |  |
|  |  |  |  |  |  |  |  |  |  |  |  |  |  |  |  |  |  |  |  |  |  |  |  |  |  |  |  |  |  |  |  |  |  |  |  |  |  |  |
| Construct | Nr of studies |  |  |  | 1 |  | 1 | 1 | 1 | 1 |  |  |  |  | 2 |  | 2 |  |  | 1 |  |  | 1 | 1 | 1 |  |  |  |  |  | 1 |  |  | 1 | 1 | 1 | 1 |  |
| validity | ROB score |  |  |  | A |  | A | D | I | I |  |  |  |  | I |  | A |  |  | D |  |  | V | A | I |  |  |  |  |  | I |  |  | I | I | I | I |  |
|  | Rating |  |  |  | + |  | + | + | - | - |  |  |  |  | + |  | + |  |  | - |  |  | + | -       ** | |  |  |  |  |  | + |  |  | + | + | + | - |  |
|  | Quality |  |  |  |  |  |  |  | VL | VL |  |  |  |  | L |  | H |  |  | VL |  |  |  | L ** | |  |  |  |  |  | VL |  |  | VL | VL | VL | VL |  |
|  |  |  |  |  |  |  |  |  |  |  |  |  |  |  |  |  |  |  |  |  |  |  |  |  |  |  |  |  |  |  |  |  |  |  |  |  |  |  |
| Convergent | Nr of studies | 1 | 1 | 1 | 1 |  | 1 | 1 | 4 | 1 | 1 |  |  |  |  |  |  |  |  |  | 5 | 1 | 4 | 2 | 2 |  |  |  |  |  | 5 | 1 | 2 |  |  |  |  |  |
| validity | ROB score | V | A | I | V |  | A | D | A | I | D |  |  |  |  |  |  |  |  |  | A | I | A | D | I |  |  |  |  |  | A | D | I |  |  |  |  |  |
|  | Rating | + | + | ? | + |  | + |  | + * * | | |  |  |  |  |  |  |  |  |  | - | ? | + ** | | |  |  |  |  |  | + | + | + |  |  |  |  |  |
|  | Quality |  |  |  |  |  |  |  | H ** | | |  |  |  |  |  |  |  |  |  | H |  | H ** | | |  |  |  |  |  | H | H |  |  |  |  |  |  |
|  |  |  |  |  |  |  |  |  |  |  |  |  |  |  |  |  |  |  |  |  |  |  |  |  |  |  |  |  |  |  |  |  |  |  |  |  |  |  |
| Criterion | Nr of studies | 1 | 1 |  |  |  |  |  | 1 | 1 | 1 |  |  |  |  |  |  |  |  |  | 1 | 1 | 1 | 1 | 1 |  |  |  |  | 1 | 1 | 1 |  |  |  |  |  |  |
| validity | ROB score | V | A |  |  |  |  |  | A | I | I |  |  |  |  |  |  |  |  |  | A | I | A | I | I |  |  |  |  | V | V | A |  |  |  |  |  |  |
|  | Rating | + | + |  |  |  |  |  | -        ** | | + |  |  |  |  |  |  |  |  |  | - |  | + | ? |  |  |  |  |  | - | + | + |  |  |  |  |  |  |
|  | Quality |  |  |  |  |  |  |  | L ** | |  |  |  |  |  |  |  |  |  |  | L |  |  | VL |  |  |  |  |  | H |  | M |  |  |  |  |  |  |
|  |  |  |  |  |  |  |  |  |  |  |  |  |  |  |  |  |  |  |  |  |  |  |  |  |  |  |  |  |  |  |  |  |  |  |  |  |  |  |
| Cross cultural validity/ | Nr of studies |  |  |  | 2 |  |  |  |  |  |  |  |  |  |  |  | 1 |  |  |  |  |  | 1 |  |  |  |  |  |  |  |  |  |  |  |  |  |  |  |
| measurement invariance | ROB score |  |  |  | I |  |  |  |  |  |  |  |  |  |  |  | I |  |  |  |  |  | I |  |  |  |  |  |  |  |  |  |  |  |  |  |  |  |
|  | Rating |  |  |  | ? |  |  |  |  |  |  |  |  |  |  |  | ? |  |  |  |  |  | ? |  |  |  |  |  |  |  |  |  |  |  |  |  |  |  |
|  | Quality |  |  |  |  |  |  |  |  |  |  |  |  |  |  |  | VL |  |  |  |  |  | VL |  |  |  |  |  |  |  |  |  |  |  |  |  |  |  |
|  |  |  |  |  |  |  |  |  |  |  |  |  |  |  |  |  |  |  |  |  |  |  |  |  |  |  |  |  |  |  |  |  |  |  |  |  |  |  |
| Known-Group | Nr of studies |  |  |  |  |  |  |  |  |  |  |  |  |  |  |  |  |  |  |  |  |  |  |  |  |  |  |  |  | 1 |  |  |  |  |  |  |  |  |
| Validity | ROB score |  |  |  |  |  |  |  |  |  |  |  |  |  |  |  |  |  |  |  |  |  |  |  |  |  |  |  |  | D |  |  |  |  |  |  |  |  |
|  | Rating |  |  |  |  |  |  |  |  |  |  |  |  |  |  |  |  |  |  |  |  |  |  |  |  |  |  |  |  | ? |  |  |  |  |  |  |  |  |
|  | Quality |  |  |  |  |  |  |  |  |  |  |  |  |  |  |  |  |  |  |  |  |  |  |  |  |  |  |  |  | L |  |  |  |  |  |  |  |  |
|  |  |  |  |  |  |  |  |  |  |  |  |  |  |  |  |  |  |  |  |  |  |  |  |  |  |  |  |  |  |  |  |  |  |  |  |  |  |  |
| Concurrent | Nr of studies |  |  |  |  |  |  |  | 6 | 3 | 5 | 1 | 1 |  |  |  |  |  |  |  |  |  | 5 | 2 |  |  |  |  |  |  |  |  |  |  |  |  |  |  |
| Validity | ROB score |  |  |  |  |  |  |  | A | A | V | I | I |  |  |  |  |  |  |  |  |  | V | M |  |  |  |  |  |  |  |  |  |  |  |  |  |  |
|  | Rating |  |  |  |  |  |  |  | + | - | + | + | - |  |  |  |  |  |  |  |  |  | + | + |  |  |  |  |  |  |  |  |  |  |  |  |  |  |
|  | Quality |  |  |  |  |  |  |  | M | M | M | L |  |  |  |  |  |  |  |  |  |  | M ** | |  |  |  |  |  |  |  |  |  |  |  |  |  |  |
|  |  |  |  |  |  |  |  |  |  |  |  |  |  |  |  |  |  |  |  |  |  |  |  |  |  |  |  |  |  |  |  |  |  |  |  |  |  |  |
| Internal | Nr of studies | 1 | 1 |  | 1 |  | 1 |  | 1 |  |  |  |  | 1 | 2 |  | 1 | 2 |  | 1 |  |  |  |  |  |  |  |  |  |  |  |  |  |  |  |  |  |  |
| consistency | ROB score | V | A |  | A |  | A |  | A |  |  |  |  | V | V |  | V | D |  | V |  |  |  |  |  |  |  |  |  |  |  |  |  |  |  |  |  |  |
|  | Rating | + | + |  | - |  | - |  | + |  |  |  |  | + | ? |  | + | + |  | + |  |  |  |  |  |  |  |  |  |  |  |  |  |  |  |  |  |  |
|  | Quality |  |  |  |  |  |  |  | L |  |  |  |  |  | H |  | L | L |  | M |  |  |  |  |  |  |  |  |  |  |  |  |  |  |  |  |  |  |
|  |  |  |  |  |  |  |  |  |  |  |  |  |  |  |  |  |  |  |  |  |  |  |  |  |  |  |  |  |  |  |  |  |  |  |  |  |  |  |
| Inter-Rater Reliability | Nr of studies | 1 |  |  | 1 | 2 | 1 |  | 4 |  |  |  |  |  | 1 |  |  |  |  | 1 | 1 |  | 2 | 2 |  |  |  |  |  |  | 4 | 2 | 1 |  |  |  |  |  |
|  | ROB score | V |  |  | D | G | A |  | I |  |  |  |  |  | D |  |  |  |  | V | D |  | D | I |  |  |  |  |  |  | I | I | D |  |  |  |  |  |
|  | Rating | + |  |  | - | - | - |  | + |  |  |  |  |  | - |  |  |  |  | + | - |  | + | - |  |  |  |  |  |  | + | ? | - |  |  |  |  |  |
|  | Quality |  |  |  |  |  |  |  | L |  |  |  |  |  | VL |  |  |  |  | M |  |  |  |  |  |  |  |  |  |  | L |  |  |  |  |  |  |  |
|  |  |  |  |  |  |  |  |  |  |  |  |  |  |  |  |  |  |  |  |  |  |  |  |  |  |  |  |  |  |  |  |  |  |  |  |  |  |  |
| Test-retest Reliability |  |  |  |  |  |  |  |  | 2 | 2 | 5 |  |  |  |  |  | 1 | 2 | 1 |  | 2 |  | 2 | 2 |  |  |  |  |  |  | 4 |  |  |  |  |  |  |  |
|  |  |  |  |  |  |  |  |  | V | A | A |  |  |  |  |  | D | A | D |  | I |  | D | I |  |  |  |  |  |  | I |  |  |  |  |  |  |  |
|  |  |  |  |  |  |  |  |  | + | + | - |  |  |  |  |  | ? | + | + |  | + |  | + | + |  |  |  |  |  |  | + |  |  |  |  |  |  |  |
|  |  |  |  |  |  |  |  |  | H | M | L |  |  |  |  |  | VL | H | L |  | L |  | VL | L |  |  |  |  |  |  | L |  |  |  |  |  |  |  |
|  |  |  |  |  |  |  |  |  |  |  |  |  |  |  |  |  |  |  |  |  |  |  |  |  |  |  |  |  |  |  |  |  |  |  |  |  |  |  |
| Measurement error | Nr of studies |  |  |  |  |  |  |  | 4 | 1 |  |  |  |  |  |  |  |  |  |  |  |  | 1 | 1 | 1 |  |  |  |  |  |  |  |  |  |  |  |  |  |
|  | ROB score |  |  |  |  |  |  |  | A | D |  |  |  |  |  |  |  |  |  |  |  |  | A | D | I |  |  |  |  |  |  |  |  |  |  |  |  |  |
|  | Rating |  |  |  |  |  |  |  | -** | |  |  |  |  |  |  |  |  |  |  |  |  | ? | -** | |  |  |  |  |  |  |  |  |  |  |  |  |  |
|  | Quality |  |  |  |  |  |  |  | H* | |  |  |  |  |  |  |  |  |  |  |  |  | M | VL** | |  |  |  |  |  |  |  |  |  |  |  |  |  |
|  |  |  |  |  |  |  |  |  |  |  |  |  |  |  |  |  |  |  |  |  |  |  |  |  |  |  |  |  |  |  |  |  |  |  |  |  |  |  |
| Responsiveness | Nr of studies | 1 |  |  | 1 | 2 | 1 | 1 | 5 | 1 | 3 | 4 | 3 |  |  |  | 1 |  |  |  | 1 | 1 | 1 | 1 | 2 | 1 | 3 | 1 | 3 |  | 1 | 3 | 1 |  |  |  |  |  |
|  | ROB score | A |  |  | A | V | D | V | D | A | I | V | A |  |  |  | A |  |  |  | D | I | V | I | D | I | D | A | I |  | D | I | D |  |  |  |  |  |
|  | Rating | + |  |  | + | + | + | + | -** | | ? | +** | |  |  |  | + |  |  |  | - | ? | + | + | -* | | -** | | ? |  | + | ? | ? |  |  |  |  |  |
|  | Quality |  |  |  |  |  |  |  | M** | | L | M** | |  |  |  | M |  |  |  |  | L |  |  |  |  | L** | | L |  |  | L | L |  |  |  |  |  |
|  |  |  |  |  |  |  |  |  |  |  |  |  |  |  |  |  |  |  |  |  |  |  |  |  |  |  |  |  |  |  |  |  |  |  |  |  |  |  |
| Sensitivity | Nr of studies |  |  |  |  |  |  |  |  |  |  |  |  |  |  | 2 |  |  |  |  |  |  |  |  |  |  |  |  |  | 1 |  |  |  |  |  |  |  |  |
|  | ROB score |  |  |  |  |  |  |  |  |  |  |  |  |  |  | A |  |  |  |  |  |  |  |  |  |  |  |  |  |  |  |  |  |  |  |  |  |  |
|  | Range (%) |  |  |  |  |  |  |  |  |  |  |  |  |  |  | 18-53 |  |  |  |  |  |  |  |  |  |  |  |  |  | 36-66 |  |  |  |  |  |  |  |  |
|  | Quality |  |  |  |  |  |  |  |  |  |  |  |  |  |  |  |  |  |  |  |  |  |  |  |  |  |  |  |  |  |  |  |  |  |  |  |  |  |
|  |  |  |  |  |  |  |  |  |  |  |  |  |  |  |  |  |  |  |  |  |  |  |  |  |  |  |  |  |  |  |  |  |  |  |  |  |  |  |
| Specificity | Nr of studies |  |  |  |  |  |  |  |  |  |  |  |  |  |  | 2 |  |  |  |  |  |  |  |  |  |  |  |  |  |  |  |  |  |  |  |  |  |  |
|  | ROB score |  |  |  |  |  |  |  |  |  |  |  |  |  |  | A |  |  |  |  |  |  |  |  |  |  |  |  |  |  |  |  |  |  |  |  |  |  |
|  | Range (%) |  |  |  |  |  |  |  |  |  |  |  |  |  |  | 77-97 |  |  |  |  |  |  |  |  |  |  |  |  |  |  |  |  |  |  |  |  |  |  |
|  | Quality |  |  |  |  |  |  |  |  |  |  |  |  |  |  |  |  |  |  |  |  |  |  |  |  |  |  |  |  |  |  |  |  |  |  |  |  |  |

*Note*: ***COSMIN Grade** **A**: scales with sufficient content validity and at least low-quality evidence for sufficient internal consistency: recommended for use; ***COSMIN Grade** **B**: scales that do not fit into either category 1) or 2): potential to be recommended for use; ***COSMIN Grade** **C**: scales with high-quality evidence indicating an insufficient measurement property: not recommended for use; Empty cells indicate no available results for measurement properties; ^1^ The positive results for the BAT / QPS should be confirmed by at least one additional study; ** The value refers to all the studies included in the highlighted box.

*Legend*:

| **BAT** - Breakthrough Pain Assessment Tool  **BAT-DL** – BAT-dutch version  **BAT**-K – BAT-korean version  **BPI** - Brief Pain Inventory  **mBPI** - Modified Brief Pain Inventory  **BPI-PS** - Pain Severity subscale of the BPI-Pain Severity subscale  **BPI-SF** - Brief Pain Inventory - Short Form  **CPI –** Cancer Pain Inventory  **CAAS** - Cardiac Analgesic Assessment Scale  **COMFORT** - Comfort Scale  **COMFORT – B** - Comfort Scale - Behavioral  **COMFORT-neo** - Comfort Scale for Neonates  **COMFORT-W** - COMFORT without blood pressure  **CPOT** – Critical-Care Pain Observation Tool  **Doloplus-2** - Doloplus Scale, version 2  **FLACC** - Face, Legs, Activity, Cry, Consolability Scale  **mFLACC** - Modified Face, Legs, Activity, Cry, Consolability Scale  **revised-FLACC** - Revised Face, Legs, Activity, Cry, Consolability Scale  **IQ-BTP** - Italian Questionnaire for Breakthrough Pain  **LANSS** - Leeds Assessment of Neuropathic Symptoms and Signs  **McGill Pain Quest.** - McGill Pain Questionnaire | **MBPS** - Modified Behavioral Pain Scale  **MAPS** - Multidimensional Assessment of Pain Scale  **DN4** – Neuropathic Pain Diagnostic Questionnaire |
| --- | --- |
|  | **NCS** - Nociception Coma Scale  **NCS-r** - Nociception Coma Scale - Revised  **NPRS** - Numeric Pain Rating Scale  **PACSLAC-II** - Pain Assessment Checklist for Seniors with Limited Ability to Communicate – II  **PES -** Pain Evaluation Scale  **PDQ –** PainDETECT screening tool  **PPP** - Pediatric Pain Profile  **QUEDI** - Questionnaire for Neuropathic Pain Identification  **QPS** - Questionnaire on Pain caused by Spasticity  **VAS** - Visual Analogue Scale  **VRS** - Verbal Rating Scale  **VRS-5** - Verbal Rating Scale, 5-point version  **VRS-16** - Verbal Rating Scale, 16-point version  **Webber's** - Webber's Breakthought Cancer Pain Algorithm  **FPS-6 -** 6 Point Faces Pain Scale  **FPS-7 -** 7 Point Faces Pain Scale  **VRS-16** - 16-Point Verbal Rating Scale  **NRS-21** - 21-Point Numeric Rating Scale  **VRS-5** - 5-Point Verbal Rating Scale |
|  |  |
